# Comprehensive Molecular and Epidemiological Characterization of *Staphylococcus aureus* Isolated from Bovine Mastitis in Water Buffalo of the Peshawar Division, Khyber Pakhtunkhwa, Pakistan

**DOI:** 10.3390/pathogens14080735

**Published:** 2025-07-25

**Authors:** Salma Javed, Jo-Ann McClure, Irfan Ullah, Shahzad Ali, Mohammad Ejaz, Sadia Tabassum, Muhammad Ali Syed, Kunyan Zhang

**Affiliations:** 1 Department of Zoology, Hazara University, Mansehra 21120, Khyber Pakhtunkhwa, Pakistan; salma.38641@gmail.com; 2 Centre for Antimicrobial Resistance, Alberta Health Services/Alberta Precision Laboratories/University of Calgary, Calgary, AB T2N 4N1, Canada; joannmarie.mcclure@albertahealthservices.ca; 3 Department of Biological Sciences, Karakorum International University, Ghizer Campus, Ghizer 15100, Gilgit Baltistan, Pakistan; irfanullah@kiu.edu.pk; 4 Wildlife Epidemiology and Molecular Microbiology Laboratory (One Health Research Group), Discipline of Zoology, Department of Wildlife & Ecology, University of Veterinary and Animal Sciences, Lahore 54000, Pakistan; shahzad.ali@uvas.edu.pk; 5 Department of Microbiology, Government Postgraduate College Mandian Abbottabad, Abbottabad 22044, Pakistan; mohammadejaz113@gmail.com; 6 Department of Microbiology, The University of Haripur, Haripur 22620, Pakistan; 7 Department of Pathology & Laboratory Medicine, University of Calgary, Calgary, AB T2N 1N4, Canada; 8 The Calvin, Phoebe and Joan Snyder Institute for Chronic Diseases, University of Calgary, Calgary, AB T2N 1N4, Canada

**Keywords:** *Staphylococcus aureus*, mastitis, molecular characterization, epidemiology, Khyber Pakhtunkhwa, antibiotic resistance

## Abstract

Water buffalo (*Bubalus bubalis*) are a primary source of milk in Pakistan, where bovine mastitis is a significant health issue among cattle, leading to substantial economic losses. *Staphylococcus aureus* is a predominant pathogen associated with mastitis; however, a detailed molecular characterization of the strains in the country remains limited. We previously characterized mastitis strains from the Hazara division of Khyber Pakhtunkhwa, Pakistan. In this study, we investigated mastitis cases in the Peshawar division, including samples from both animals and human farm workers for comparison. Higher rates of mastitis (67.27% of animals) and sub-clinical mastitis (91.03% of positive animals) were identified in Peshawar than for those (34.55% and 75.31%, respectively) previously observed in Hazara. Methicillin-susceptible *S. aureus* (MSSA) belonging to clonal complex 9 (ST2454) were predominant. Methicillin-resistant *S. aureus* (MRSA) belonging to ST22 and ST8 were also detected in the Nowshera district. While no *S. aureus* colonization was observed among animal handlers, evidence of hand contamination suggests a potential route for pathogen spread. Low levels of antibiotic resistance were noted amongst isolates, but higher rates were seen in MRSA. This study presents only the second comprehensive molecular investigation of *S. aureus* isolated from buffalo mastitis in Pakistan and indicates a concerning rise in mastitis within the province.

## 1. Introduction

Water buffalo (*Bubalus bubalis*) are a major source of milk in many countries, including in Pakistan, where they account for approximately 68% of total milk production [1]. Bovine mastitis, an inflammatory disease of the mammary gland parenchyma, is one of the most prevalent diseases in cattle, leading to considerable economic loss due to treatment costs, loss in milk production, dumping of contaminated milk, and an elevated culling rate of diseased animals [2]. Mastitis can present as either a clinical or sub-clinical infection, with clinical disease characterized by sudden onset, alterations of milk composition, decreased production, and inflammation of mammary quarters [3]. Sub-clinical disease, on the other hand, shows no visible changes to udders or milk quality; however, it is marked by decreased milk production and increased somatic cell counts [3]. Sub-clinical infection can persist for long periods of time and spread rapidly though herds, making it 15–40 times more common than clinical infection [4]. *Staphylococcus aureus* is one of the agents associated with intramammary infection in buffalo; in Pakistan it accounts for approximately 46% of mastitis cases [5]. Infection with *S. aureus* can result in biofilm formation, which contributes to immune evasion and chronic infection [6]. Infection with *S. aureus* can also lead to acute mastitis, characterized by inflammation and mammary tissue damage, often resulting from the production of virulence factors, such as enterotoxins, toxic shock syndrome toxin-1, exfoliative toxins, and hemolysins [7].

Several studies examining the prevalence and epidemiology of mastitis have been performed in Pakistan; however, until recently, detailed molecular characterization of staphylococci associated with the disease did not exist. In 2022, we characterized *S. aureus* from bovine mastitis in the Hazara division of Khyber Pakhtunkhwa, the first study to provide comprehensive molecular details about mastitis-related *S. aureus* in the country [8]. The results showed that *S. aureus* was isolated from 18.4% (81/440) of animals and, as expected, sub-clinical mastitis (75.3%; 61/81) was more prevalent than clinical disease (24.7%; 20/81). A total of 19.6% (20/102) of isolates were methicillin resistant *S. aureus* (MRSA), and strains from three clonal complexes were identified, including CC9 (spa type t7286 and t7867), CC101 (t2078), and CC22 (t8934). Antibiotic resistance levels were low overall, although rates were higher in the MRSA than in the methicillin sensitive *S. aureus* (MSSA). To expand upon this knowledge, the current study examines the epidemiology and molecular characteristics of *S. aureus* in a second division of the Khyber Pakhtunkhwa (KP) province of Pakistan, the Peshawar division. While the Hazara region is the wettest part of Pakistan and is cooler than other regions because of its altitude, the Peshawar region has a hot semi-arid climate, possibly altering *S. aureus* epidemiology in the area.

## 2. Materials and Methods

### 2.1. Study Area and Sample Collection

**Figure 1:**
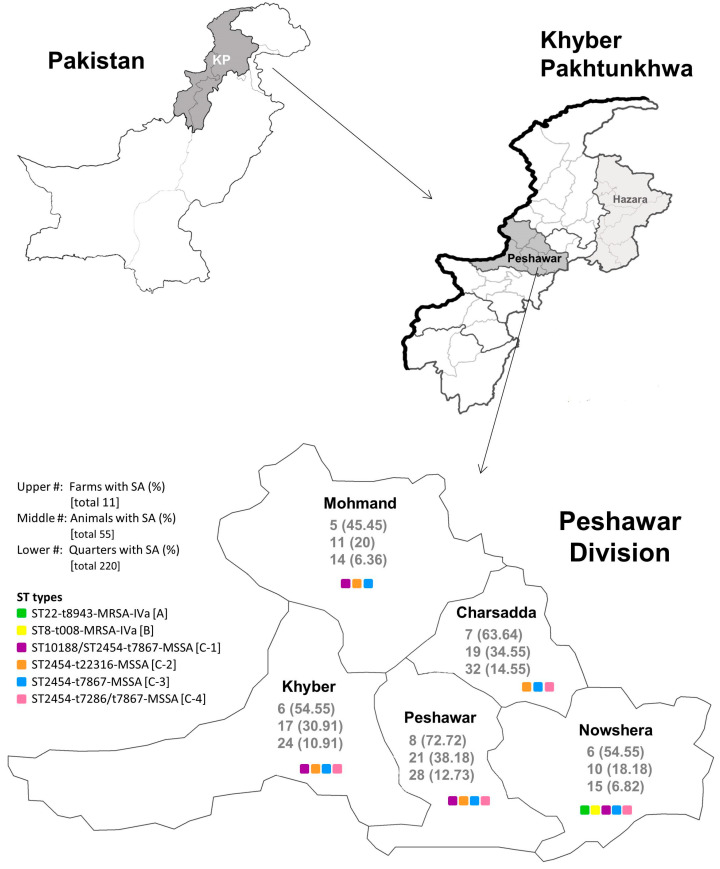
Pakistan and its provinces, with their corresponding divisions and districts. The province of Khyber Pakhtunkhwa is shown shaded in grey in the northwest corner of the Pakistan map. Within KP, the Hazara division from our last study is shown in light grey, and the Peshawar division from this study is shown in medium grey. The five districts of the Peshawar division are shown in the lowest map, along with their corresponding frequency of *S. aureus* infection at the farm, animal, and quarter level, as well as the sequence types identified in each district. Note: #, number; %, percentage; SA, *S. aureus*.

The present study was conducted in the Peshawar Division of Khyber Pakhtunkhwa (KP), Pakistan (September 2020–March 2022). Pakistan is divided into four administrative provinces and one federal territory. The province of Khyber Pakhtunkhwa is in the northwestern part of the country (see Figure 1), and is further divided into 7 divisions, including the Hazara division, targeted in our previous study, and the Peshawar division from this study. The Peshawar division is itself divided into 5 districts, including the Charsadda, Khyber, Mohmand, Nowshera, and Peshawar districts, all of which were included in this study. Prior to sampling, a questionnaire was used to collect information about all the farms and buffalo from which milk was collected for the study. Details such as shelter, vaccination status, feed, treatment, milk condition, milk color, body weight, duration of lactation, udder shape, teat length, teat shape, teat lesion, and cord formation were obtained. Eleven buffalo farms were selected from each district of the Peshawar division, chosen with the criteria that they be at least 5 km apart (all were 5–10 km), and must contain at least 5 animals. A total of 5 milking water buffalo were conveniently/randomly selected for sampling from each farm, for a total of 275 buffalo (any breed). Both symptomatic (clinical mastitis) and asymptomatic (sub-clinical or uninfected) animals were included within the sampling.

Milk samples were collected from all four teats (quarters) of each of the 275 water buffalo. The length of each of teat was measured (up to 1 mm accuracy) using a ruler, and its diameter at the base, midpoint, and apex were measured with a Vernier caliper. Prior to collection, teats were cleaned with cotton soaked with 70% ethanol, then the first few drops of milk discarded. A surf field mastitis test (SFMT) with 4% surf solution (Excel, Unilever, Karachi, Pakistan) was used to test the next few drops of milk for mastitis infection. Samples that formed clumps from increased somatic cell counts (SCC) were considered positive and were collected in sterile 15 mL falcon tube, while non-clumping samples were considered negative and were discarded. Positive samples were transported on ice to the microbiology lab of the Department of Microbiology at the University of Haripur for further analysis.

Samples were also collected from a single farm worker on each of the farms that was included in the study, for a total of 55 workers. The animal handler who was most frequently in contact with the animals for feeding and milking was chosen, and their skin (surface of the thumb tip on the right hand), nose (anterior naris of either the right or left naris of the nose), and ear (outer part of the ear canal of either the right or left ear) were swabbed with sterile cotton swabs moistened with 0.9% NaCl solution. Swabs were transported to the microbiology lab of the Department of Microbiology at the University of Haripur for analysis.

### 2.2. Bacterial Cultivation and Susceptibility Testing of S. aureus Strains

Fifty microlitres of each milk sample was spread onto mannitol salt agar (MSA) plates and incubated at 37 °C for 24 h. For the human samples, swabs were streaked directly onto the MSA plates, then incubated. *S. aureus* was identified using standard microbiology tests: isolates that fermented mannitol, were Gram-positive cocci; isolates that produced DNase and catalase and were tube coagulase positive were designated *S. aureus* [9].

*S. aureus* was screened for resistance to 16 antibiotics by disc diffusion assays following standard Clinical and Laboratory Standards Institute guidelines, 34th edition [10]. Antibiotics assessed include ampicillin (AMP), amoxicillin (AMX), lincomycin (LCM), ceftazidime (CAZ), azithromycin (AZM), ceftriaxone (CFO), norfloxacin (NOR), cefoxitin (FOX), gentamycin (GEN), erythromycin (ERY), tetracycline (TET), doxycycline (DOX), clindamycin (CLI), trimethoprim/sulfamethoxazole (SXT), rifampin (RIF), and linezolid (LZD).

### 2.3. S. aureus Molecular Characterization

DNA for molecular analysis was extracted from *S. aureus* using the previously described rapid boiling method [11]. The identity of *S. aureus* was confirmed using a multiplex polymerase chain reaction (PCR) assay capable of distinguishing coagulase negative staphylococci from SA, while simultaneously differentiating MRSA from MSSA, and identifying the Panton–Valentine leukocidin (PVL) genes [12]. Strain C2406 (CP095933) was used as a positive control. Pulsed field gel electrophoresis (PFGE) was used to fingerprint the strains following digestion with SmaI (New England Biolabs, Ipswich, MA, USA), as previously described by [13]. Canadian (CMRSA1-10) and USA (USA100-800) reference strains were included for comparison. BioNumerics Ver. 6.6 (Applied Maths, Sint-Martens-Lattem, Belgium) was used to analyse the DNA fingerprints, using a position tolerance of 1.5 as well as an optimization of 0. Strains were further characterized for the presence of antiseptic resistance genes [14], as well as SCC*mec* [15], and accessory gene regulator (*agr*) [16]. Sequencing was performed to identify the staphylococcal protein A (*spa*) [17] and multilocus sequence types (MLST) [18], with analysis performed using the Center for Genomic Epidemiology spaTyper 1.0 [19] and PubMLST databases [20].

### 2.4. Ethics Approval

The Ethics and Research Committee of the Department of Zoology, Hazara University, Mansehra, Pakistan (Reference No. hu. zool. rerc321; approval date: 8 May 2019) approved this study.

## 3. Results

### 3.1. Overall Epidemiology and Molecular Characteristics of Staphylococcus aureus from Water Buffalo Milk Samples in Peshawar Division

**Figure 2:**
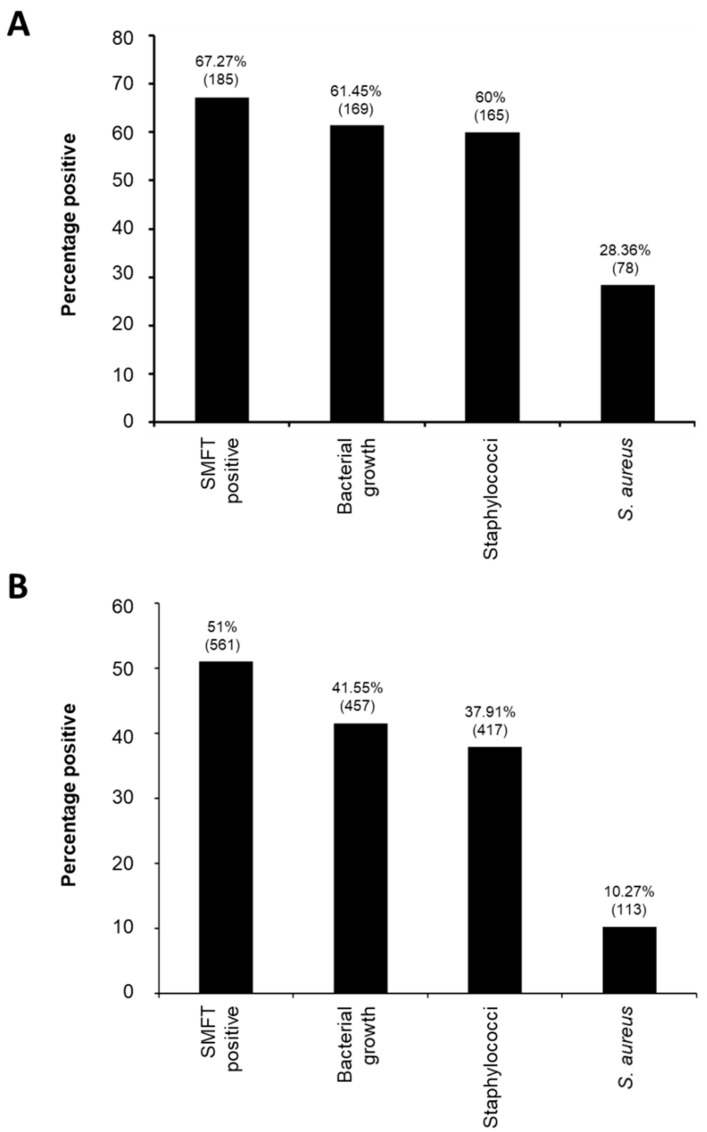
Frequency of bovine mastitis infection in the Peshawar division. (**A**) The proportion of animals that were SFMT positive, produced bacterial growth, had staphylococci present, and contained *S. aureus*. (**B**) The proportion of quarters that were SFMT positive, produced bacterial growth, had staphylococci present, and contained *S. aureus* are shown. In both situations the percentage is shown above the graph, with the corresponding number in brackets.

A total of 275 milking water buffalo were included in the current study, of which 185 (67.27%) were SMFT positive, indicating the presence of infection. As seen in Figure 2A, bacteria were cultured in 169 (61.45%) of the samples, 165 (60%) were determined to carry staphylococci, 143 (52%) were mannitol fermenting, and 78 (28.36%) were *S. aureus* positive. With 4 quarters on each of the 275 milking buffalo, a total of 1100 quarters were tested. As seen in Figure 2B, 561 (51%) of the quarters were SFMT positive, from which 457 (41.55%) resulted in bacterial growth, 417 (37.91%) contained staphylococci, 321 (29.18%) were mannitol fermenting, and 113 (10.27%) were positive for *S. aureus*. In *S. aureus* infected animals, 49 (43.36%) of the cases involved infection in only one quarter, while in 23 (20.35%) of the cases involved infection in two quarters, and 6 (5.31%) cases involved infection in three quarters. No animals were infected in all four quarters. With respect to the specific quarter infected, there was an almost equal distribution between infection in the left front (*n* = 30, 26.5%), left rear (*n* = 31, 27.4%), right front (*n* = 23, 20.4%), and right rear (*n* = 29, 25.7%) quarters.

In this study, samples were collected from 55 farms. Bovine mastitis was detected on 32 (58.18%) of the farms, with both clinical and sub-clinical infection identified on seven of them, and the remaining 25 farms only having sub-clinically infected animals. Amongst the 78 *S. aureus* positive animals, 7 (8.97%) displayed clinical infection, and amongst the 113 infected quarters, 17 (15.04%) were present on clinically infected animals.

Molecular characterization of the *S. aureus* isolates (summarized in Table 1) showed that MSSA was present on 29 (90.63%) of the 32 farms, all of which had animals with sub-clinical infection, while 7 of the farms also had clinical infection present. Similarly, MRSA was detected on 5 (15.63%) of the 32 farms, all of which had animals with sub-clinical infection, while 1 farm also had clinical infection present. Of the 78 *S. aureus* positive animals, 73 (93.59%) carried MSSA, 66 (90.41%) of which were from sub-clinical infection, and 7 (9.59%) of which were from a clinical infection. Likewise, 8 (10.26%) for the 78 *S. aureus* positive animals carried MRSA, 1 of which was a clinical infection while the 7 seven were sub-clinical infections. At the quarters level, 104 (92.04%) of the 113 infected quarters carried MSSA, while 9 (7.96%) carried MRSA.

### 3.2. Epidemiology of S. aureus from Milk Samples in Each District of the Peshawar Division

The incidence of *S. aureus* related mastitis per farm, animal, and quarter in each district of the Peshawar division is shown in Figure 1 and Table 2, as well as the breakdown between clinical and sub-clinical infection. Briefly, in Peshawar, *S. aureus* was detected in eight (72.72%) farms, 21 (38.18%) animals, and 28 (12.73%) quarters. In Charsadda, *S. aureus* was detected in seven (63.64%) farms, 19 (34.55%) animals, and 32 (14.55%) quarters. In Nowshera, *S. aureus* was detected in six (54.55%) farms, 10 (18.18%) animals, and 15 (6.82%) quarters. In Khyber, *S. aureus* was detected in six (54.55%) farms, 17 (30.91%) animals, and 24 (10.91%) quarters. In Mohmand, *S. aureus* was detected in five (45.45%) farms, 11 (20%) animals, and 14 (6.36%) quarters. In each district, sub-clinical infection was more prevalent than clinical infection, as summarized in Table 2.

Initial molecular typing of the *S. aureus* isolated from milk samples in each district indicated that MRSA was only isolated from buffalo in the Nowshera district, while all samples from the other districts were exclusively (100%) MSSA (see Figure 1 and Table 2). In the Nowshera district, MRSA was isolated from five (83.33%) of the *S. aureus* positive farms, while MSSA was isolated from three (50%) of the farms. On two (33.33%) of those farms, both MRSA and MSSA were isolated. At the animal level, eight (80%) of the *S. aureus* positive animals in the Nowshera district had mastitis caused by MRSA, while five (50%) had mastitis caused by MSSA. Three (30%) of those animals, on two farms, were infected by both MRSA and MSSA. At the quarter level, MRSA was isolated from nine (60%) of the *S. aureus* infected quarters, while MSSA was isolated from six (40%) of them. No quarters had both MRSA and MSSA co-infection. In all districts sub-clinical infection predominated, both amongst MSSA and MRSA infected animals.

### 3.3. Detailed Molecular Characteristics of S. aureus Isolated from Bovine Mastitis Milk Samples

**Figure 3:**
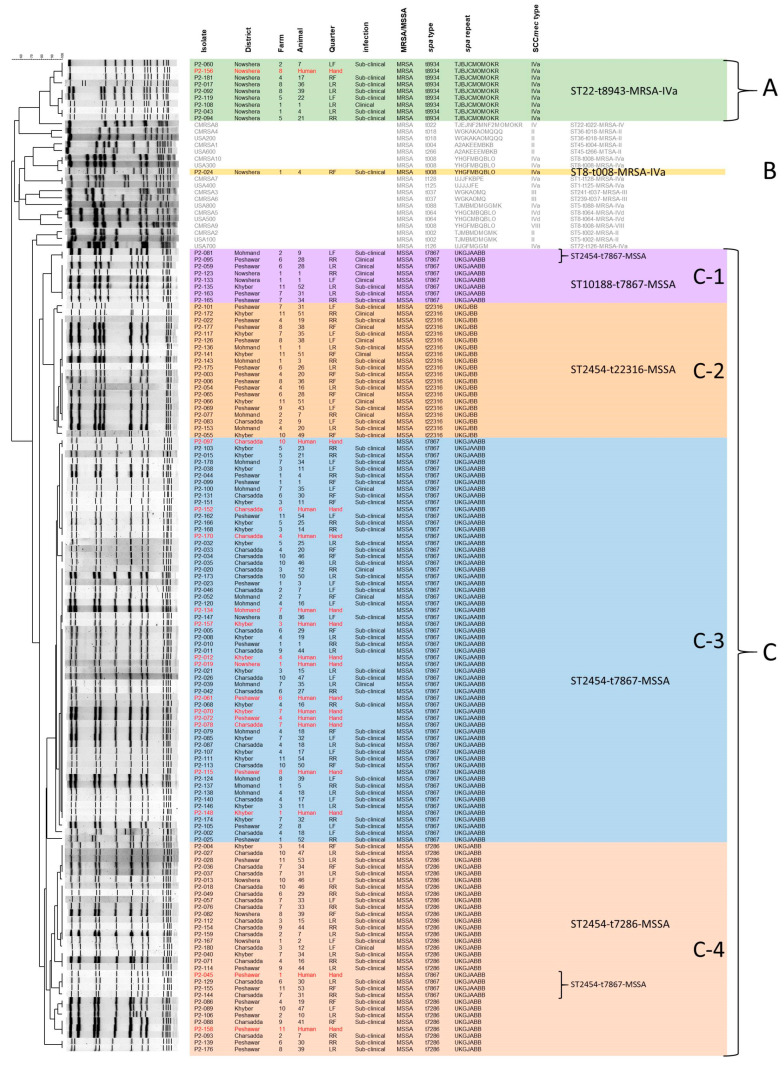
Pulsed field gel electrophoresis fingerprints for all *S. aureus* isolated in the Peshawar Division of Pakistan. Division, farm, animal, and quarter information are indicated, as well as infection status of the animal and molecular characteristics of the isolates. Group A isolates (ST22-t8943-MRSA-IVa) are shaded in green, group B (ST8, t008-MRSA-IVa) in yellow, subgroup C-1 (ST2454/ST10188-t7867-MSSA) in purple, subgroup C-2 (ST2454-t22316-MSSA) in peach, subgroup C-3 (ST2454-t7867-MSSA) in blue, and subgroup C-4 (ST2454-t7286/t7867-MSSA) in pink. Reference strain types from USA and Canada (CMRSA) are indicated in grey font. Isolates from human caregivers are indicated in red font. Notes: *spa*, staphylococcal protein A; SCC*mec*, Staphylococcal cassette chromosome *mec*; LF, left front; LR, left rear; RF, right front; RR, right rear.

Molecular characterization indicated that MSSA accounted for the majority of *S. aureus* isolated from mastitis cases in the Peshawar division. One hundred and four (92.04%) of the *S. aureus* were MSSA, while only nine (7.96%) were MRSA. A PFGE analysis showed that the strains fell into three groups, A, B and C, as seen in Figure 3. Group A (green shading) was composed of eight buffalo isolates sharing greater than 88.45% similarity in the Dice coefficient of correlation (DCC); group B (yellow shading) was composed of a single isolate matching the USA300 PFGE type, and the remaining isolates fell into group C. Group C can itself be broken down into 4 subgroups; subgroup C-1 (purple shading) had 8 isolates sharing greater than 89.76% similarity in the DCC; C-2 (pink shading) had 20 isolates sharing greater than 92.32% similarity in the DCC; C-3 (blue shading) had 47 buffalo isolates sharing greater than 85.17% similarity in the DCC; and C-4 (pink shading) had 29 buffalo isolates sharing greater than 83.09% similarity in the DCC. Within each group there was a mixture of both clinical and sub-clinical infection present; however, groups A and B comprised exclusively MRSA isolates, while group C comprised solely MSSA.

More detailed typing concurred with the PFGE groupings. All isolates in group A were found to belong to MLST type ST22, *spa* type t8943 (TJBJCMOMOKR), and accessory gene regulator type I (*agrI*), and to carry SCC*mec* IVa (ST22-t8943-MRSA-IVa). The lone isolate in group B belonged to MLST type ST8, *spa* type t008 (YHGFMBQBLO), and *agrI*, and to carry SCC*mec* IVa (ST8-t008-MRSA-IVa). This isolate also carried the PVL, *arcA*, and phi-4 bacteriophage marker genes, identifying it as USA300. Group C isolates all belonged to ST2454 (3-3-1-1-264-1-10), or its single locus variant ST10188 (3-3-41-1-264-1-10), which differs from ST2454 by a single nucleotide in the *glpF* locus, and all were *agr* type II. Two isolates in subgroup C-1 belonged to ST2454-t7867(UKGJAABB)-MSSA, while the other six isolates were ST10188-t7867-MSSA. All isolates in subgroup C-2 were ST2454-t22316(UKGJBB)-MSSA, and all isolates in subgroup C-3 belonged to ST2454-t7867-MSSA. Subgroup C-4 contained three isolates that were ST2454-t7867-MSSA, while the remaining isolates were ST2454-t7286(UKGJABB)-MSSA. None of the isolates carried the multidrug efflux pump genes (*qacA* or *qacB*), small multidrug resistance genes (*smr*), or mupirocin resistance genes (*mupA* or *mupB*).

Examining the data on a districtwide basis (Figure 1 and Figure 3 and Table S1), we see that all strains in group A were isolated from buffalo in the Nowshera district, as was the isolate in group B. Strains in group C came from buffalo in all five districts, however, we did note differences between the subgroups. The majority of isolates from the Charsadda division fell into subgroup C-3 and C-4, with none from Charsadda in C-1, and only one from the region in C-2. None of the isolates from the Mohmand district fell into subgroup C-4. Similarly, no subgroup C-2 strains were from the Nowshera district, and only one isolate in subgroup C-3 came from the district.

Interestingly, in 18 instances, more than one strain group was isolated from a single buffalo, as shown by boxes around the PFGE groups in Table S1. Seven animals from five farms in the Charsadda district carried both ST2454-t7867-MSSA (subgroup C-3) and ST2454-t7286-MSSA (subgroup C-4), while one animal from a sixth farm in Charsadda carried both ST2454-t7286-MSSA (subgroup C-4) and ST2454-t7867-MSSA (subgroup C-4). One animal from one farm in the Khyber district carried both ST2454-t7867-MSSA (subgroup C-3) and ST2454-t7286-MSSA (subgroup C-4), one animal from one farm in the Mohmand district carried both ST2454-t22316-MSSA (subgroup C-2) and ST2454-t7867-MSSA (subgroup C-3). In the Peshawar district, there were four instances on four different farms where two different MSSA were isolated from a single animal, including one with ST2454-t22316-MSSA (subgroup C-2) and ST2454-t7286-MSSA (subgroup C-4), two with both ST10188-t7867-MSSA (subgroup C-1) and ST2454-t22316-MSSA (subgroup C-2), and one with both ST2454-t7286-MSSA (subgroup C-4) and ST2454-t7867-MSSA (subgroup C-4). In the Nowshera district, we identified one buffalo infected by ST22-t8934-MRSA-IVa (group A) and ST10188-t7867-MSSA (subgroup C-1). A second animal from the same farm was infected by both ST22-t8934-MRSA-IVa (group A) and ST8-t008-MRSA-IVa (group B). Finally, two animals from the same farm in Nowshera were co-infected with MRSA and MSSA, one with both ST22-t8934-MRSA-IVa (group A) and ST2454-t7867-MSSA (subgroup C-3), and one with both ST22-t8934-MRSA-IVa (group A) and ST2454-t7286-MSSA (subgroup C-4).

### 3.4. Epidemiological Investigation on the Zoonotic Transmission of S. aureus Strains from Bovine Mastitis Infected Water Buffalo to Their Caretakers

In the present study, swabs were taken from the skin (hands), anterior nares, and outer ear canal of one caretaker on each farm in the study, for a total of 165 samples from 55 personnel. No *S. aureus* positive samples were obtained from the ears or nose of any personnel; however, 16 (29.09%) of the hand samples were *S. aureus* positive, as seen in Table 3 and Table S1. One worker carried MRSA on their hands, while the remaining fifteen carried MSSA. In the Peshawar district, five (45.45%) of the caretakers carried *S. aureus* on their milking hands, while in both the Charsadda and Khyber districts, four (36.36%) caretakers carried it, in the Nowshera district, two (18.18%) caretakers carried it (including the only MRSA), and in the Mohmand district, only one (9.09%) caretaker carried *S. aureus* on their hands.

In the Charsadda, Khyber, and Mohmand districts, all human samples belonged to ST2454-t7867-MSSA (subgroup C-3), matching strains found in buffalo on the corresponding farms. In the Nowshera district, one human sample belonged to ST22-t8934-MRSA-IVa (group A), matching buffalo isolates from the same farm; however, the other human isolate belonged to ST2454-t7867-MSSA (subgroup C-3), which was not isolated from any buffalo on that farm. In the Peshawar district, strain type ST2454-t7286-MSSA (subgroup C-4) was isolated from a caretaker, matching a buffalo isolate on the same farm. On another farm in the Peshawar district, strain ST2454-t7867-MSSA (subgroup C-4) was isolated from a caretaker, but that strain group was not isolated from any animals on the corresponding farm. Similarly, strain ST2454-t7867-MSSA (subgroup C-3) was isolated from three farm workers in the Peshawar district, but the strain group was not found in any animals on the corresponding farms.

### 3.5. Antibiotic Resistance of S. aureus Isolated from Milk Samples in the Peshawar District

Resistance of all *S. aureus* isolated in the Peshawar division to 16 antibiotics was determined, as shown in Table 4 and Table S2. Overall, resistance of animal isolates to the antibiotics was low, with the exception to ceftazidime (74.34%), amoxicillin (23.89%), and ampicillin (21.24%). MRSA showed high levels of resistance to all of the antibiotics with all nine (100%) resistant to ceftazidime, eight of nine (88.89%) resistant to ampicillin, cefoxitin, clindamycin, amoxicillin, and ceftriaxone, seven of nine (77.78%) resistant to gentamicin, lincomycin, azithromycin, and erythromycin, and six of nine (66.67%) resistant to tetracycline and norfloxacin. MRSA showed lower resistance to doxycycline, rifampin, trimethoprim-sulfamethoxazole (four of nine; 44.44% each), and linezolid (one of nine; 11.11%). Strain P2-108 is exceptional in that it is MRSA (Figure S1 for molecular confirmation), yet sensitive to everything except ceftazidime (R) and erythromycin (I). SCC*mec* typing indicated that this strain carries a type IVa SCC*mec* cassette (Figure 3). In addition, sequencing of the *mecA* gene confirmed it is intact and undisrupted (Figure S2). Resistance patterns for human isolates matched the resistance patterns seen in the animal isolates, with the sole human MRSA having higher resistance to antibiotics than MSSA.

In general, resistance to the antibiotics was very low in Charsadda, Khyber, Mohmand, and Peshawar (Table 4), though higher levels were noted in each region for amoxicillin and ceftazidime, and a higher level of resistance was also seen to ampicillin (35.71%) and trimethoprim-sulfamethoxazole (21.43%) in Mohmand. Resistance to all antibiotics was higher in the Nowshera district, attributable to MRSA, and it is the only district where resistance was observed to all 16 antibiotics. It is noteworthy that resistance to norfloxacin was only seen in the Nowshera district, resistance to clindamycin was only seen in Charsadda and Nowshera, resistance to doxycycline was only seen in Mohmand and Nowshera, resistance to linezolid was only seen in Nowshera and Peshawar, and resistance to ceftriaxone was only seen in Khyber and Nowshera.

## 4. Discussion

Mastitis is a significant disease challenging the dairy industry on a global scale, particularly serious in Pakistan as it is a limiting factor in the development of the country’s dairy industry. Research has shown that mastitis accounts for 17% of the economic loss from animal disease, and 80% of sub-clinical mastitis cases are due to *Staphylococcus aureus* [21,22]. As *S. aureus* is the major causative agents of the disease, we examined the epidemiology and genetic characteristics of *S. aureus* causing mastitis in water buffalo in the Peshawar division of Khyber Pakhtunkhwa, Pakistan, and compared the results to a previous study we did in the Hazara division of the same province.

In this study, 67.27% of the animals and 51% of the quarters in the Peshawar division were found to have bovine mastitis, which represents a higher rate than we identified in our previous study in the Hazara division (mastitis was identified in 34.55% of animals and 34.03% of quarters in that study) [8]. These rates are also higher than those found in a 2013 study conducted in the Peshawar district, in which 36.35% of buffalo in rural regions were suffering from mastitis [23]; however, high rates like ours have been described in the Tehsil Gojra district of Punjab, Pakistan, where 60.27% of buffalo were found to have mastitis [24]. Likewise, in a 2021 study by Ali et al. the prevalence of mastitis in buffalo was found to range from 57–80% in four districts of the Peshawar division, with staphylococci being the most prevalent pathogen, isolated from 34% of cases [25]. Rates of *S. aureus* infection in the current study were similar to those noted by Ali et al., with our results showing 28.36% of the animals and 10.27% of the quarters infected with the species. Again, these rates are higher than we identified in the Hazara division, where only 18.41% of animals and 5.8% of quarters were infected with *S. aureus*. A study by Khan et al. also found that rates of *S. aureus* mastitis were highest in the Peshawar–Mardan division (30%), while they were lowest in the Hazara division (16%), in line with our numbers [26]. Looking district by district, the incidence of *S. aureus* mastitis in this study ranged from a low of 18.18% of animals in the Nowshera district, to a high of 38.18% of animals in the Peshawar district. Comparable data from other studies for districtwide rates is limited, but in the Peshawar district, *S. aureus* mastitis rates of 34.88% (Kundi buffalo) and 40.42% (Nili Ravi buffalo) have been documented, in keeping with our findings [23]. Similar to our previous study in the Hazara division, and to other published studies in the country, sub-clinical mastitis was more prevalent than clinical mastitis in the Peshawar division. In this study, however, the rates of sub-clinical mastitis (91.03% of *S. aureus* positive animals, 84.96% of positive quarters) were notably higher than we found in Hazara (75.31% of *S. aureus* positive animals, 68.63% of quarters) or have been found in other parts of the country, such as Punjab (67.3%) or Lahore (59.64%) [27,28]. This data could suggest that mastitis rates are increasing in the province; however, differential climate and husbandry practices (such as shelter type, farm area, and sanitization), as well as differential infection control practices and the use of antibiotics, could contribute to the different rates noted in each study. Sub-clinical mastitis can spread stealthily through a herd on the hands of workers, contaminated equipment, or farm environment, and with such a low rate of animals with visible signs of infection to trigger a response, the high numbers seen in this study likely point to a need for enhanced vigilance and stricter adherence to infection control practices.

As with our previous investigations in the Hazara division, detailed molecular characterization of *Staphylococcus aureus* isolated from bovine mastitis in the Peshawar division was not available prior to this study. Overall, MSSA (seen in 93.59% of positive animals, 92.04% of positive quarters) was more commonly encountered than MRSA (seen in 10.26% of positive animals, 7.96% of positive quarters), with MRSA only identified in the Nowshera district. While low levels of MRSA positivity from mastitis are noted in locations such as Germany (16.7% of milk samples), India (13.1% of Sahiwal cattle), Korea (6.3% in dairy cows), and Wisconsin (1.8% of dairy cows) [29,30,31,32], higher rates have been reported in Pakistan. In the Islamabad and Rawalpindi districts of the Pothohar region, 21% of *S. aureus* from milk samples were MRSA, and in the Faisalabad district of Punjab 38% of *S. aureus* from buffalo milk were MRSA [7,33]. In the Hazara division, our previous study also identified a higher MRSA prevalence than seen here, with 19.6% of *S. aureus* identified as MRSA. It is particularly interesting that four of the five districts from the Peshawar division had an MRSA prevalence of 0%, yet high rates of MRSA (as high as 74.4%) have been documented in human hospital samples in the region [34,35,36]. The human samples include inpatient and outpatient cultures of pus (particularly from patients with skin abscesses), blood, urine, HVS swabs, catheter tips, tissue, and implants ranging from 2012 to 2024. This could indicate that there is minimal transmission of MRSA from humans to animals; however, without a more detailed patient history, it is difficult to draw conclusions, as the general population would not be expected to have significant contact with farm animals. The reason that MRSA was not detected in four of the districts in this study remains unclear, as it has been isolated from mastitis cases in Peshawar in the past [37], and there were no specific infection control or husbandry practices that we are aware of that would contribute.

All *S. aureus* from the region underwent genetic typing, showing that they belonged to one of three major strain groups: ST2454-MSSA (either t7867, t7286, or t22316) and the very closely related ST10188-t7867-MSSA, ST22-t8934-MRSA-IVa, or ST8-t008-MRSA-IVa. The majority of isolates (n = 104, 92%), and all the MSSA, were ST2454 or the single locus variant ST10188, which both belong to clonal complex nine (CC9). This is the same lineage identified as being dominant in our previous study in the Hazara division and is commonly encountered in nearby countries. Sivakumar et al. reported ST2454-t7867 and ST2454-t7286 as the most frequent *S. aureus* isolated from bovine mastitis in several states of India [38]. Similarly, Annamanedi et al. identified CC9-ST2454-t7867 as the most frequent isolated sequence type of *S. aureus* from bovine mastitis in the Uttar Pradesh, Telangana, Meghalaya, and Maharashtra states of India [39]. In Bangladesh, Rahman et al. also identified ST2454-t7867 in raw milk samples obtained from mastitis cows [40]. To date, there have been no reports of ST10188 in mastitis cows; however, it only differs from ST2454 by a single base pair change in the *glpF* locus and likely represents a mutation of an existing strain in the region. Likewise, there have been no reports of t22316 (UKGJBB) in bovine mastitis, which only differs from t7286 (UKGJABB) by a single repeat, and t7867 (UKGJAABB) by two repeats. It is interesting to note that, in some buffalo, we saw co-infection with two different MSSA with different *spa* types (t7867 and t7286), or co-infection with two MSSA with the same *spa* type but from different PFGE groups. In every case, infection with each organism was in separate quarters, never occurring together within one quarter. Bovine quarters are generally considered separate compartments; therefore, it is possible for them to become independently infected with different strains. It is also possible that a buffalo was infected by a single strain, which later mutated within the animal. Spa types t7867 and t7286 differ by only the loss of a single repeat. Evolution of the *spa* region has been described following long-term persistence on *S. aureus* in cystic fibrosis patients, with mutational events occurring in 10% of the strains analysed [41]. They concluded that every 93 months deletions or duplications occurred in the *spa* repeats, thought to be caused by slipped-strand mispairing combined with inadequate DNA mismatch repair systems. With the high rates of “hidden” sub-clinical mastitis infection occurring in buffalo in the region, it is possible that long term colonization of animals is occurring and resulting in *spa* gene mutation, leading to the apparent presence of multiple strain groups within a single animal. Further studies involving long term sampling of animals would be needed to address this issue.

Notably absent from this study was the strain group ST101-t2078-MSSA, which was identified in 14.6% of the samples in the Hazara division, isolated from five of the eight districts in the study. ST101 staphylococci are known to colonize and to cause disease in both humans and animals globally, and to be present in bulk milk samples [42,43,44,45]; however, the strain group does not appear to be relevant on the farms in this division, despite its presence in the province. Amongst the MRSA in Peshawar, 88.9% of them belonged to ST22-t8943-MRSA-IVa, which was the only strain group that we previously identified for MRSA in the Hazara division. CC22 MRSA (such as ST22-t2986-MRSA) have been identified in humans in Pakistan, however they differ from the ST22 in this study [46,47], again, suggesting that transmission of MRSA between humans and animals is not occurring. This study differed from our previous one in that here, for the first time, we isolated ST8-t008-MRSA-IVa from a buffalo with sub-clinical mastitis. ST8-MRSA-USA300 has been isolated from bulk tank milk on dairy farms in Minnesota, USA [48], but there is little evidence that it is a significant pathogen causing mastitis in buffalo in Pakistan. USA300 MRSA have been identified in clinical samples in Pakistan [49,50], suggesting that MRSA significant to human disease could occasionally be transmitting to buffalo in the region, albeit at a very low rate.

To address the issue of zoonotic transmission between handlers and the buffalo, we swabbed the nose, ear, and hands of workers on each farm, data that was not collected in our previous study in Hazara. Zoonotic transmission of *S. aureus* between bovines and their handlers has been documented in Algeria and India [51,52]; however, none of the caretakers in this study carried *S. aureus* in their nares or ear canals, indicating that they were not colonized and unknowingly acting as a source of transmission. On 16 of the farms (50% of the *S. aureus* positive farms), *S. aureus* was isolated from the hands of farm workers, with 10 farms having the strain group isolated from the buffalo and human hands matching. This indicates that there is often cross contamination of farm workers, which can become a significant source of pathogen transmission between animals. With the high rates of sub-clinical infection noted in this study, this becomes even more significant as there are no obvious signs that animals are infectious, and disease can rapidly spread on the hands of workers. On six farms, the *S. aureus* strain type on workers’ hands did not match the types isolated from cows with mastitis, although they were consistent with strain groups found on other farms in the region. Four instances, in particular, were located in the Peshawar district. Farms in this study are small scale and located in close proximity (5–10 km), sometimes with the exchange of people or animals between them. Contamination of the workers could have come from animals on other farms, or from the environment; however, it appears that adherence to infection control practices has successfully prevented the spread of disease on these farms. This study is limited by the low number of human isolates obtained, therefore expanded sampling of farm workers would be needed to determine if our conclusions are correct.

Antibiotics represent a commonly employed method of infection control in Pakistan, both for treatment and for prevention of disease. A study in Lahore, Pakistan, found that 90% of farmers considered self-medication more economical than consulting a veterinarian, and thus 22% of farmers do not consult a vet for prescribing antibiotics, and 32.9% do not follow correct dosage instructions [53]. Antibiotic use in cattle in Pakistan is higher than international averages, with beta-lactams, aminoglycosides, and tetracyclines most commonly chosen [54,55]. This incorrect usage of antimicrobials can contribute to the development of antibiotic resistance amongst mastitis pathogens, impacting therapeutic options in the future, and possibly spreading through the environment to human populations. The *S. aureus* isolates in our study (both from animals and humans) had generally low levels of resistance to antibiotics, though had higher resistance to ceftazidime (74.34%), amoxicillin (23.89%), and ampicillin (21.24%). High rates of resistance to ceftazidime (100%), amoxicillin (29%), and ampicillin (42%) were also noted in our previous study in the Hazara division and, in both studies, resistance levels to every antibiotic except linezolid was higher amongst MRSA than MSSA. The two studies differed in that, in the Hazara division, there was a higher rate of resistance (26.47%) to lincomycin, while in the Peshawar division, only 8.85% of the *S. aureus* were resistant. High levels of lincomycin resistance (84–98%) have been documented in raw milk samples from Pakistan [25,56], therefore the differences noted in our two studies likely point to a less frequent use of the antibiotic in Peshawar than Hazara. Antibiotic usage data was not collected for this study, however, so it remains difficult to make a definitive conclusion on the issue. Lastly, it is worth noting that one of the MRSA isolates in this study (P2-108) carries a type IVa SCC*mec* cassette (Figure 3) with an intact and undisrupted *mecA* gene (Figures S1 and S2), yet displays an unusual antibiotic resistance profile, being susceptible to most antibiotics tested and resembling the pattern typically observed in MSSA. Global regulators, such as SarA (staphylococcal accessory regulator A) and MarR (multiple antibiotic resistance regulator), are known to control expression of approximately 350 genes, and impact expression of genes involved in resistance mechanisms [57,58,59]. Further studies would be needed to fully understand the cause.

This study presents the first detailed molecular characterization of *S. aureus* isolated from bovine mastitis cases in the Peshawar division of Pakistan. Our findings revealed similar epidemiological and molecular patterns as observed in our previous research conducted in the Hazara division, but with a higher prevalence seen in the current study. Notably, sampling of farm workers indicated that, while human colonization does not appear to play a major role in disease transmission, hand contamination is common and may serve as a potential source of spread. The high prevalence of sub-clinical mastitis observed underscores the urgent need to strengthen infection control measures to mitigate the growing economic impact of this disease.

## Figures and Tables

**Table 1:** Epidemiology of *S. aureus* related bovine mastitis cases at the farm, animal, and quarter level.

	Farm No (%)	Animal No (%)	Quarter No (%)
***S. aureus* positive**	32 (58.18)	78 (28.36)	113 (10.27)
**Sub-Clinical**	32 (100.00)	71 (91.03)	96 (84.96)
**Clinical**	7 (21.88)	7 (8.97)	17(15.04)
**MSSA**	29 (90.63)	73 (93.59)	104 (92.04)
**Sub-Clinical**	29 (100.00)	66 (90.41)	88 (84.62)
**Clinical**	7 (24.14)	7 (9.59)	16 (15.38)
**MRSA**	5 (15.63)	8 (10.26)	9 (7.96)
**Sub-Clinical**	5 (100.00)	7 (87.50)	8 (88.89)
**Clinical**	1 (20.00)	1 (12.50)	1 (11.11)

Note: No., number; %, percentage; total farms = 55; total animals = 275; total quarters = 1100.

**Table 2:** Epidemiology and molecular characteristics of *S. aureus* isolates in each district of the Peshawar division.

	District				
	Charsadda No. (%)	Khyber No. (%)	Mohmand No. (%)	Nowshera No. (%)	Peshawar No. (%)
**Farm**					
***S. aureus* positive**	7 (63.64)	6 (54.55)	5 (45.45)	6 (54.55)	8 (72.72)
**MSSA**	7 (100)	6 (100)	5 (100)	3 (50)	8 (100)
**Sub-clinical**	7 (100)	6 (100)	5 (100)	3 (100)	8 (100)
**Clinical**	1 (14.29)	1 (16.67)	2 (40)	1 (33.33)	2 (25.00)
**MRSA**	0	0	0	5 (83.33)	0
**Sub-clinical**	0	0	0	5 (100)	0
**Clinical**	0	0	0	1 (20)	0
**Animal**					
***S. aureus* positive**	19 (34.55)	17 (30.91)	11 (20)	10 (18.18)	21 (38.18)
**MSSA**	19 (100)	17 (100)	11 (100)	5 (50)	21 (100)
**Sub-clinical**	18 (94.74)	16 (94.12)	9 (81.82)	4 (80)	19 (90.48)
**Clinical**	1 (5.26)	1 (5.88)	2 (18.18)	1 (20)	2 (9.52)
**MRSA**	0	0	0	8 (80)	0
**Sub-clinical**	0	0	0	7 (87.5)	0
**Clinical**	0	0	0	1 (12.5)	0
**Quarter**					
***S. aureus* positive**	32 (14.55)	24 (10.91)	14 (6.36)	15 (6.82)	28 (12.73)
**MSSA**	32 (100)	24 (100)	14 (100)	6 (40)	28 (100)
**Sub-clinical**	30 (93.75)	21 (87.50)	10 (71.43)	4 (66.67)	23 (82.14)
**Clinical**	2 (6.25)	3 (12.50)	4 (28.57)	2 (33.33)	5 (17.86)
**MRSA**	0	0	0	9 (60)	0
**Sub-clinical**	0	0	0	8 (88.89)	0
**Clinical**	0	0	0	1 (11.11)	0

Note: No., number; %, percentage. In each district: total farms = 11; total animals = 55; total quarters = 220.

**Table 3:** Strain types identified in human and mastitis samples within the same farm.

District	Farm	Human Isolate (PFGE Group)	Mastitis Isolates (FPGE Group)
Charsadda	4	ST2454-t7867-MSSA (C-3) *	ST2454-t7867-MSSA (C-3) * ST2454-t7286-MSSA (C-4)
	6	ST2454-t7867-MSSA (C-3) *	ST2454-t7867-MSSA (C-3) * ST2454-t7286-MSSA (C-4)
	7	ST2454-t7867-MSSA (C-3)	ST2454-t7867-MSSA (C-4) ST2454-t7286-MSSA (C-4)
	10	ST2454-t7867-MSSA (C-3) *	ST2454-t7867-MSSA (C-3) * ST2454-t7286-MSSA (C-4)
Khyber	3	ST2454-t7867-MSSA (C-3) *	ST2454-t7867-MSSA (C-3) * ST2454-t7286-MSSA (C-4)
	4	ST2454-t7867-MSSA (C-3) *	ST2454-t7867-MSSA (C-3) *
	7	ST2454-t7867-MSSA (C-3) *	ST2454-t7867-MSSA (C-3) * ST2454-t7286-MSSA (C-4) ST2454-t22316-MSSA (C-2)
	11	ST2454-t7867-MSSA (C-3) *	ST2454-t7867-MSSA (C-3) * ST2454-t22316-MSSA (C-2) ST2454-t7867-MSSA (C-1)
Mohmand	7	ST2454-t7867-MSSA (C-3) *	ST2454-t7867-MSSA (C-3) *
Nowshera	1	ST2454-t7867-MSSA (C-3)	ST2454-t7867-MSSA (C-1) ST22-t8934-MRSA (A) ST2454-t7286-MSSA (C-4) ST8-t008-MRSA (B)
	8	ST22-t8934-MRSA (A) *	ST22-t8934-MRSA (A) * ST2454-t7867-MSSA (C-3) ST2454-t7286-MSSA (C-4)
Peshawar	1	ST2454-t7867-MSSA (C-4)	ST2454-t7867-MSSA (C-3)
	4	ST2454-t7867-MSSA (C-3)	ST2454-t22316-MSSA (C-2) ST2454-t7286-MSSA (C-4)
	6	ST2454-t7867-MSSA (C-3)	ST2454-t22316-MSSA (C-2) ST10188-t7867-MSSA (C-1) ST2454-t7867-MSSA (C-1) ST2454-t7286-MSSA (C-4)
	8	ST2454-t7867-MSSA (C-3)	ST2454-t22316-MSSA (C-2) ST2454-t7286-MSSA (C-4)
	11	ST2454-t7286-MSSA (C-4) *	ST2454-t7286-MSSA (C-4) * ST2454-t7867-MSSA (C-3) ST2454-t7867-MSSA (C-4)

Note: *, human and animal samples are the same strain type.

**Table 4:** Overall and districtwide resistance of all animal-related *S. aureus* isolates to 16 antibiotics.

		District					
		Overall %	Charsadda %	Khyber %	Mohmand %	Nowshera % (MRSA)	Peshawar %
**Ampicillin**	**S**	78.76	84.38	91.67	64.29	46.67 (11.11)	85.71
	**I**	0	0	0	0	0	0
	**R**	21.24	15.63	8.33	35.71	53.33 (88.89)	14.28
**Cefoxitin**	**S**	92.92	100	100	100	46.67 (11.11)	100
	**I**	0	0	0	0	0	0
	**R**	7.08	0	0	0	53.33 (88.89)	0
**Clindamycin**	**S**	80.53	84.38	87.5	92.86	40 (11.11)	85.71
	**I**	9.73	6.25	12.5	7.14	6.67 (0)	14.29
	**R**	9.73	9.38	0	0	53.33 (88.89)	0
**Gentamycin**	**S**	78.76	84.38	87.5	78.57	33.33 (11.11)	89.29
	**I**	10.62	9.38	8.33	14.29	20.00 (11.11)	7.14
	**R**	10.62	6.25	4.17	7.14	46.67 (77.78)	3.57
**Amoxicillin**	**S**	76.11	81.25	79.17	78.57	40 (11.11)	85.71
	**I**	0	0	0	0	0	0
	**R**	23.89	18.75	20.83	21.43	60 (88.89)	14.28
**Doxycycline**	**S**	80.53	90.63	83.33	78.57	40 (22.22)	89.29
	**I**	14.16	9.38	16.67	14.29	26.67 (33.33)	10.71
	**R**	5.31	0	0	7.14	33.33 (44.44)	0
**Lincomycin**	**S**	80.53	84.38	91.67	64.29	46.67 (11.11)	92.86
	**I**	10.62	12.5	8.33	21.43	6.67 (11.11)	7.14
	**R**	8.85	3.13	0	14.29	46.67 (77.78)	0
**Ceftazidime**	**S**	0	0	0	0	0	0
	**I**	25.66	34.38	37.5	28.57	6.67 (0)	14.29
	**R**	74.34	65.63	62.5	71.43	93.33 (100)	85.71
**Rifampin**	**S**	77.88	81.25	83.33	64.29	40 (22.22)	96.43
	**I**	16.81	18.75	12.5	28.57	33.33 (33.33)	3.57
	**R**	5.31	0	4.17	7.14	26.67 (44.44)	0
**Trimethoprim-sulfamet-hoxazole**	**S**	79.65	87.5	83.33	64.29	53.33 (22.2)	89.29
	**I**	11.50	9.38	8.33	14.29	20 (33.33)	10.71
	**R**	8.85	3.13	8.33	21.43	26.67 (44.44)	0
**Linezolid**	**S**	92.04	93.75	95.83	92.86	80 (66.67)	21.43
	**I**	6.19	6.25	4.17	7.14	13.33 (22.22)	3.57
	**R**	1.77	0	0	0	6.67 (11.11)	3.57
**Azithromycin**	**S**	84.07	84.38	95.83	92.86	46.67 (11.11)	89.29
	**I**	7.08	9.38	4.17	7.14	6.67 (11.11)	7.14
	**R**	8.85	6.25	0	0	46.67 (77.78)	3.57
**Ceftriaxone**	**S**	76.99	84.38	75	78.57	33.33 (11.11)	92.86
	**I**	15.04	15.63	20.83	21.43	13.33 (0)	7.14
	**R**	7.96	0	4.17	0	53.33 (88.89)	0
**Tetracycline**	**S**	81.42	93.75	87.5	78.57	26.67 (11.11)	92.86
	**I**	9.73	3.13	8.33	14.29	26.67 (22.22)	7.14
	**R**	8.85	3.13	4.17	7.14	46.67 (66.67)	0
**Norfloxacin**	**S**	87.61	93.75	91.67	92.86	46.67 (11.11)	96.43
	**I**	7.08	6.25	8.33	7.14	13.33 (22.22)	3.57
	**R**	5.31	0	0	0	40 (66.67)	0
**Erythromycin**	**S**	69.91	78.13	79.17	64.29	33.33 (0)	75
	**I**	14.16	6.25	16.67	21.43	20 (22.22)	14.29
	**R**	15.93	15.63	4.17	14.29	46.67 (77.78)	10.71

## Data Availability

The original contributions presented in this study are included in the article/Supplementary Materials. Further inquiries can be directed to the corresponding author.

## Supplementary Materials

The following supporting information can be downloaded at: https://www.mdpi.com/article/10.3390/pathogens14080735/s1, Table S1: Characteristics of all *S. aureus* isolated from buffalo with mastitis in the Peshawar division of Pakistan; Table S2: Antibiotic resistance patterns for all *S. aureus* isolated from buffalo mastitis cases; Figure S1: Multiplex PCR assay confirming the presence of *mecA* in strain P2-108. The original DNA extract, a second MRSA from the same strain type (P2-119) and a MSSA (P2-123) were tested, along with the re-cultured/re-isolated sample of P2-108; Figure S2: DNA and corresponding amino acid sequences for *mecA* in strain P2-108.
