# Supplementary material for: Comprehensive Molecular and Epidemiological Characterization of Staphylococcus aureus Isolated from Bovine Mastitis in Water Buffalo of the Peshawar Division, Khyber Pakhtunkhwa, Pakistan
Source: Pathogens. 2025 Jul 25;14(8):735. doi: 10.3390/pathogens14080735 (PMC12388927; doi:10.3390/pathogens14080735)
Supplement: Supplementary file 1 [file pathogens-14-00735-s001.zip › Table S1.pdf]

Table S1. Characteristics of all *S. aureus* isolated from buffaloes with mastitis in the Peshawar division of Pakistan.

Sample	District	Farm	Animal	Quarter	Disease	Strain	*spa* type†	MLST type‡	*agr* type	PFGE group
P2-46	Charsadda	2	7	LF	Subclinical	MSSA	t7867	ST2454	II	C-3
P2-159	Charsadda	2	7	LR	Subclinical	MSSA	t7286	ST2454	II	C-4
P2-93	Charsadda	2	7	RR	Subclinical	MSSA	t7286	ST2454	II	C-4
P2-83	Charsadda	2	9	LF	Subclinical	MSSA	t22316	ST2454	II	C-2
P2-180	Charsadda	3	12	LF	Clinical	MSSA	t7286	ST2454	II	C-4
P2-20	Charsadda	3	12	RR	Clinical	MSSA	t7867	ST2454	II	C-3
P2-112	Charsadda	3	15	LR	Subclinical	MSSA	t7286	ST2454	II	C-4
P2-170	Charsadda	4	*hand	-	-	MSSA	t7867	ST2454	II	C-3
P2-71	Charsadda	4	16	RR	Subclinical	MSSA	t7286	ST2454	II	C-4
P2-140	Charsadda	4	17	LF	Subclinical	MSSA	t7867	ST2454	II	C-3
P2-2	Charsadda	4	18	LF	Subclinical	MSSA	t7867	ST2454	II	C-3
P2-87	Charsadda	4	18	LR	Subclinical	MSSA	t7867	ST2454	II	C-3
P2-33	Charsadda	4	20	RF	Subclinical	MSSA	t7867	ST2454	II	C-3
P2-152	Charsadda	6	*hand	-	-	MSSA	t7867	ST2454	II	C-3
P2-42	Charsadda	6	27	RR	Subclinical	MSSA	t7867	ST2454	II	C-3
P2-5	Charsadda	6	29	RF	Subclinical	MSSA	t7867	ST2454	II	C-3
P2-49	Charsadda	6	29	RR	Subclinical	MSSA	t7286	ST2454	II	C-4
P2-129	Charsadda	6	30	LR	Subclinical	MSSA	t7867	ST2454	II	C-4
P2-131	Charsadda	6	30	RF	Subclinical	MSSA	t7867	ST2454	II	C-3
P2-78	Charsadda	7	*hand	-	-	MSSA	t7867	ST2454	II	C3
P2-37	Charsadda	7	31	LR	Subclinical	MSSA	t7286	ST2454	II	C-4
P2-144	Charsadda	7	31	RR	Subclinical	MSSA	t7867	ST2454	II	C-4
P2-57	Charsadda	7	33	LF	Subclinical	MSSA	t7286	ST2454	II	C-4
P2-76	Charsadda	7	33	RR	Subclinical	MSSA	t7286	ST2454	II	C-4
P2-36	Charsadda	7	34	RF	Subclinical	MSSA	t7286	ST2454	II	C-4
P2-88	Charsadda	9	41	RF	Subclinical	MSSA	t7286	ST2454	II	C-4
P2-11	Charsadda	9	44	LR	Subclinical	MSSA	t7867	ST2454	II	C-3
P2-154	Charsadda	9	44	RR	Subclinical	MSSA	t7286	ST2454	II	C-4
P2-97	Charsadda	10	*hand	-	-	MSSA	t7867	ST2454	II	C-3
P2-35	Charsadda	10	46	LR	Subclinical	MSSA	t7867	ST2454	II	C-3
P2-34	Charsadda	10	46	RF	Subclinical	MSSA	t7867	ST2454	II	C-3
P2-18	Charsadda	10	46	RR	Subclinical	MSSA	t7286	ST2454	II	C-4
P2-26	Charsadda	10	47	LF	Subclinical	MSSA	t7867	ST2454	II	C-3
P2-27	Charsadda	10	47	LR	Subclinical	MSSA	t7286	ST2454	II	C-4

P2-173	Charsadda	10	50	LR	Subclinical	MSSA	t7867	ST2454	II	C-3
P2-113	Charsadda	10	50	RF	Subclinical	MSSA	t7867	ST2454	II	C-3
P2-157	Khyber	3	*hand	-	-	MSSA	t7867	ST2454	II	C-3
P2-38	Khyber	3	11	LF	Subclinical	MSSA	t7867	ST2454	II	C-3
P2-146	Khyber	3	11	LR	Subclinical	MSSA	t7867	ST2454	II	C-3
P2-151	Khyber	3	11	RF	Subclinical	MSSA	t7867	ST2454	II	C-3
P2-4	Khyber	3	14	RF	Subclinical	MSSA	t7286	ST2454	II	C-4
P2-168	Khyber	3	14	RR	Subclinical	MSSA	t7867	ST2454	II	C-3
P2-21	Khyber	3	15	LR	Subclinical	MSSA	t7867	ST2454	II	C-3
P2-12	Khyber	4	*hand	-	-	MSSA	t7867	ST2454	II	C-3
P2-68	Khyber	4	16	RR	Subclinical	MSSA	t7867	ST2454	II	C-3
P2-107	Khyber	4	17	LF	Subclinical	MSSA	t7867	ST2454	II	C-3
P2-8	Khyber	4	19	LR	Subclinical	MSSA	t7867	ST2454	II	C-3
P2-15	Khyber	5	21	RR	Subclinical	MSSA	t7867	ST2454	II	C-3
P2-103	Khyber	5	23	RR	Subclinical	MSSA	t7867	ST2454	II	C-3
P2-32	Khyber	5	25	LR	Subclinical	MSSA	t7867	ST2454	II	C-3
P2-166	Khyber	5	25	RR	Subclinical	MSSA	t7867	ST2454	II	C-3
P2-70	Khyber	7	*hand	-	-	MSSA	t7867	ST2454	II	C-3
P2-85	Khyber	7	32	LF	Subclinical	MSSA	t7867	ST2454	II	C-3
P2-174	Khyber	7	32	RR	Subclinical	MSSA	t7867	ST2454	II	C-3
P2-40	Khyber	7	34	LR	Subclinical	MSSA	t7286	ST2454	II	C-4
P2-117	Khyber	7	35	LF	Subclinical	MSSA	t22316	ST2454	II	C-2
P2-89	Khyber	10	47	LF	Subclinical	MSSA	t7286	ST2454	II	C-4
P2-55	Khyber	10	49	RF	Subclinical	MSSA	t22316	ST2454	II	C-2
P2-148	Khyber	11	*hand	-	-	MSSA	t7867	ST2454	II	C-3
P2-66	Khyber	11	51	LF	Clinical	MSSA	t22316	ST2454	II	C-2
P2-141	Khyber	11	51	RF	Clinical	MSSA	t22316	ST2454	II	C-2
P2-172	Khyber	11	51	RR	Clinical	MSSA	t22316	ST2454	II	C-2
P2-135	Khyber	11	52	LR	Subclinical	MSSA	t7867	ST2454	II	C-1
P2-111	Khyber	11	54	RR	Subclinical	MSSA	t7867	ST2454	II	C-3
P2-136	Mohmand	1	1	LR	Subclinical	MSSA	t22316	ST2454	II	C-2
P2-143	Mohmand	1	3	RR	Subclinical	MSSA	t22316	ST2454	II	C-2
P2-137	Mohmand	1	5	RR	Subclinical	MSSA	t7867	ST2454	II	C-3
P2-52	Mohmand	2	7	RF	Clinical	MSSA	t7867	ST2454	II	C-3
P2-77	Mohmand	2	7	RR	Clinical	MSSA	t22316	ST2454	II	C-2
P2-81	Mohmand	2	9	LF	Subclinical	MSSA	t7867	ST2454	II	C-1
P2-120	Mohmand	4	16	LF	Subclinical	MSSA	t7867	ST2454	II	C-3
P2-138	Mohmand	4	18	LR	Subclinical	MSSA	t7867	ST2454	II	C-3
P2-79	Mohmand	4	18	RF	Subclinical	MSSA	t7867	ST2454	II	C-3

P2-153	Mohmand	4	20	LR	Subclinical	MSSA	t22316	ST2454	II	C-2
P2-134	Mohmand	7	*hand	-	-	MSSA	t7867	ST2454	II	C-3
P2-178	Mohmand	7	34	LF	Subclinical	MSSA	t7867	ST2454	II	C-3
P2-100	Mohmand	7	35	LF	Clinical	MSSA	t7867	ST2454	II	C-3
P2-39	Mohmand	7	35	LR	Clinical	MSSA	t7867	ST2454	II	C-3
P2-124	Mohmand	8	39	LF	Subclinical	MSSA	t7867	ST2454	II	C-3
P2-19	Nowshera	1	*hand	-	-	MSSA	t7867	ST2454	II	C-3
P2-133	Nowshera	1	1	LF	Clinical	MSSA	t7867	ST10188	II	C-1
P2-108	Nowshera	1	1	LR	Clinical	MRSA	t8934	ST22	I	A
P2-123	Nowshera	1	1	RR	Clinical	MSSA	t7867	ST10188	II	C-1
P2-167	Nowshera	1	2	LF	Subclinical	MSSA	t7286	ST2454	II	C-4
P2-43	Nowshera	1	4	LR	Subclinical	MRSA	t8934	ST22	I	A
P2-24	Nowshera	1	4	RF	Subclinical	MRSA	t008	ST8	I	B
P2-60	Nowshera	2	7	LF	Subclinical	MRSA	t8934	ST22	I	A
P2-181	Nowshera	4	17	RF	Subclinical	MRSA	t8934	ST22	I	A
P2-94	Nowshera	5	21	RR	Subclinical	MRSA	t8934	ST22	I	A
P2-119	Nowshera	5	22	LF	Subclinical	MRSA	t8934	ST22	I	A
P2-156	Nowshera	8	*hand	-	-	MRSA	t8934	ST22	I	A
P2-147	Nowshera	8	36	LF	Subclinical	MSSA	t7867	ST2454	II	C-3
P2-17	Nowshera	8	36	LR	Subclinical	MRSA	t8934	ST22	I	A
P2-92	Nowshera	8	39	LR	Subclinical	MRSA	t8934	ST22	I	A
P2-82	Nowshera	8	39	RF	Subclinical	MSSA	t7286	ST2454	II	C-4
P2-13	Nowshera	10	46	LF	Subclinical	MSSA	t7286	ST2454	II	C-4
P2-45	Peshawar	1	*hand	-	-	MSSA	t7867	ST2454	II	C-4
P2-99	Peshawar	1	1	RF	Subclinical	MSSA	t7867	ST2454	II	C-3
P2-10	Peshawar	1	1	RR	Subclinical	MSSA	t7867	ST2454	II	C-3
P2-23	Peshawar	1	3	LF	Subclinical	MSSA	t7867	ST2454	II	C-3
P2-44	Peshawar	1	4	RR	Subclinical	MSSA	t7867	ST2454	II	C-3
P2-105	Peshawar	2	8	LF	Subclinical	MSSA	t7867	ST2454	II	C-3
P2-106	Peshawar	2	10	LR	Subclinical	MSSA	t7286	ST2454	II	C-4
P2-72	Peshawar	4	*hand	-	-	MSSA	t7867	ST2454	II	C-3
P2-54	Peshawar	4	16	LR	Subclinical	MSSA	t22316	ST2454	II	C-2
P2-86	Peshawar	4	19	RF	Subclinical	MSSA	t7286	ST2454	II	C-4
P2-22	Peshawar	4	19	RR	Subclinical	MSSA	t22316	ST2454	II	C-2
P2-3	Peshawar	4	20	RF	Subclinical	MSSA	t22316	ST2454	II	C-2
P2-61	Peshawar	6	*hand	-	-	MSSA	t7867	ST2454	II	C-3
P2-175	Peshawar	6	26	LR	Subclinical	MSSA	t22316	ST2454	II	C-2

P2-59	Peshawar	6	28	LR	Clinical	MSSA	t7867	ST10188	II	C-1
P2-65	Peshawar	6	28	RF	Clinical	MSSA	t22316	ST2454	II	C-2
P2-95	Peshawar	6	28	RR	Clinical	MSSA	t7867	ST2454	II	C-1
P2-139	Peshawar	6	30	RR	Subclinical	MSSA	t7286	ST2454	II	C-4
P2-101	Peshawar	7	31	LF	Subclinical	MSSA	t22316	ST2454	II	C-2
P2-163	Peshawar	7	31	LR	Subclinical	MSSA	t7867	ST10188	II	C-1
P2-165	Peshawar	7	34	RR	Subclinical	MSSA	t7867	ST10188	II	C-1
P2-115	Peshawar	8	*hand	-	-	MSSA	t7867	ST2454	II	C-3
P2-6	Peshawar	8	36	RF	Subclinical	MSSA	t22316	ST2454	II	C-2
P2-126	Peshawar	8	38	LF	Clinical	MSSA	t22316	ST2454	II	C-2
P2-177	Peshawar	8	38	RF	Clinical	MSSA	t22316	ST2454	II	C-2
P2-176	Peshawar	8	39	LR	Subclinical	MSSA	t7286	ST2454	II	C-4
P2-69	Peshawar	9	43	LF	Subclinical	MSSA	t22316	ST2454	II	C-2
P2-114	Peshawar	9	44	LR	Subclinical	MSSA	t7286	ST2454	II	C-4
P2-158	Peshawar	11	*hand	-	-	MSSA	t7286	ST2454	II	C-4
P2-25	Peshawar	11	52	RR	Subclinical	MSSA	t7867	ST2454	II	C-3
P2-28	Peshawar	11	53	LR	Subclinical	MSSA	t7286	ST2454	II	C-4
P2-155	Peshawar	11	53	RF	Subclinical	MSSA	t7867	ST2454	II	C-4
P2-162	Peshawar	11	54	LF	Subclinical	MSSA	t7867	ST2454	II	C-3

Note: *, samples were isolated from human caregivers; †, *spa* type t7867 (UKGJAABB), t7286 (UKGJABB), t22316 (UKGJBB), t008 (YHGFMBQBLO), t8934 (TJBJCMOMOKR); ‡, ST2454 (3-3-1-1-264-1-10), ST10188 (3-3-41-1-264-1-10), ST8 (3-3-1-1-4-4-3), ST22 (7-6-1-5-8-8-6); -, not applicable; boxes indicated animals with mixed infection.
