# Supplementary material for: Comprehensive Molecular and Epidemiological Characterization of Staphylococcus aureus Isolated from Bovine Mastitis in Water Buffalo of the Peshawar Division, Khyber Pakhtunkhwa, Pakistan
Source: Pathogens. 2025 Jul 25;14(8):735. doi: 10.3390/pathogens14080735 (PMC12388927; doi:10.3390/pathogens14080735)
Supplement: Supplementary file 1 [file pathogens-14-00735-s001.zip › Table S2.pdf]

Table S2. Antibiotic resistance patterns for all *S. aureus* isolated from buffalo mastitis cases.

	Antibiotic zone of inhibition in mm (resistance)															
**Isolate**	**AMP**	**FOX**	**CLI**	**GEN**	**AMX**	**DOX**	**LCM**	**CAZ**	**RIF**	**SXT**	**LZD**	**AZM**	**CFO**	**TET**	**NOR**	**ERY**
P2-46	38 (S)	26 (S)	29 (S)	24 (S)	35 (S)	25 (S)	28 (S)	13 (R)	27 (S)	23 (S)	32 (S)	23 (S)	26 (S)	28 (S)	21 (S)	32 (S)
P2-159	30 (S)	30 (S)	26 (S)	15 (S)	37 (S)	20 (S)	31 (S)	16 (R)	30 (S)	25 (S)	34 (S)	25 (S)	28 (S)	24 (S)	28 (S)	25 (S)
P2-93	30 (S)	25 (S)	23 (S)	17 (S)	32 (S)	16 (S)	19 (I)	11 (R)	23 (S)	19 (S)	28 (S)	20 (S)	23 (S)	21 (S)	18 (S)	24 (S)
P2-83	36 (S)	31 (S)	30 (S)	25 (S)	39 (S)	24 (S)	27 (S)	14 (R)	28 (S)	27 (S)	31 (S)	28 (S)	30 (S)	28 (S)	30 (S)	32 (S)
P2-180	27 (R)	23 (S)	13 (R)	14 (I)	23 (R)	16 (S)	18 (I)	5 (R)	17 (I)	12 (I)	27 (S)	17 (I)	18 (I)	20 (S)	20 (S)	13 (R)
P2-20	25 (R)	22 (S)	17 (I)	15 (S)	21 (R)	18 (S)	14 (R)	7 (R)	19 (I)	10 (R)	25 (I)	13 (R)	19 (I)	21 (S)	16 (I)	11 (R)
P2-112	33 (S)	26 (S)	27 (S)	18 (S)	36 (S)	19 (S)	26 (S)	16 (R)	25 (S)	22 (S)	33 (S)	25 (S)	25 (S)	22 (S)	26 (S)	27 (S)
P2-170*	42 (S)	37 (S)	33 (S)	30 (S)	36 (S)	27 (S)	30 (S)	18 (I)	37 (S)	24 (S)	39 (S)	25 (S)	31 (S)	33 (S)	26 (S)	35 (S)
P2-71	31 (S)	23 (S)	25 (S)	22 (S)	35 (S)	21 (S)	25 (S)	15 (R)	27 (S)	24 (S)	30 (S)	22 (S)	23 (S)	26 (S)	23 (S)	26 (S)
P2-140	35 (S)	28 (S)	26 (S)	17 (S)	32 (S)	18 (S)	26 (S)	12 (R)	24 (S)	20 (S)	28 (S)	20 (S)	27 (S)	23 (S)	28 (S)	30 (S)
P2-2	42 (S)	33 (S)	33 (S)	23 (S)	38 (S)	22 (S)	31 (S)	18 (I)	32 (S)	26 (S)	34 (S)	27 (S)	30 (S)	25 (S)	31 (S)	35 (S)
P2-87	40 (S)	31 (S)	30 (S)	27 (S)	39 (S)	24 (S)	29 (S)	17 (I)	29 (S)	29 (S)	31 (S)	25 (S)	29 (S)	29 (S)	29 (S)	32 (S)
P2-33	32 (S)	27 (S)	25 (S)	16 (S)	36 (S)	21 (S)	24 (S)	14 (R)	26 (S)	23 (S)	33 (S)	24 (S)	24 (S)	25 (S)	23 (S)	26 (S)
P2-152*	33 (S)	31 (S)	30 (S)	35 (S)	37 (S)	30 (S)	34 (S)	20 (I)	35 (S)	39 (S)	28 (S)	21 (S)	32 (S)	37 (S)	25 (S)	29 (S)
P2-42	38 (S)	30 (S)	27 (S)	20 (S)	36 (S)	22 (S)	27 (S)	16 (R)	31 (S)	25 (S)	30 (S)	21 (S)	30 (S)	27 (S)	25 (S)	33 (S)
P2-5	33 (S)	26 (S)	28 (S)	22 (S)	38 (S)	27 (S)	29 (S)	10 (R)	27 (S)	28 (S)	32 (S)	28 (S)	25 (S)	31 (S)	29 (S)	26 (S)
P2-49	35 (S)	29 (S)	31 (S)	23 (S)	33 (S)	18 (S)	25 (S)	15 (R)	24 (S)	20 (S)	28 (S)	22 (S)	27 (S)	23 (S)	20 (S)	30 (S)
P2-129	41 (S)	29 (S)	32 (S)	25 (S)	40 (S)	23 (S)	31 (S)	21 (I)	33 (S)	27 (S)	35 (S)	29 (S)	28 (S)	26 (S)	30 (S)	31 (S)
P2-131	39 (S)	31 (S)	29 (S)	19 (S)	35 (S)	20 (S)	29 (S)	19 (I)	28 (S)	22 (S)	33 (S)	24 (S)	30 (S)	22 (S)	27 (S)	33 (S)
P2-78*	34 (S)	26 (S)	24 (S)	22 (S)	38 (S)	20 (S)	26 (S)	17 (I)	23 (S)	23 (S)	31 (S)	22 (S)	20 (I)	23 (S)	19 (S)	27 (S)
P2-37	34 (S)	28 (S)	27 (S)	21 (S)	36 (S)	15 (I)	22 (S)	12 (R)	31 (S)	25 (S)	34 (S)	26 (S)	26 (S)	18 (I)	17 (S)	28 (S)
P2-144	30 (S)	24 (S)	24 (S)	16 (S)	35 (S)	17 (S)	28 (S)	9 (R)	29 (S)	23 (S)	29 (S)	23 (S)	22 (S)	21 (S)	19 (S)	19 (I)
P2-57	36 (S)	27 (S)	29 (S)	27 (S)	39 (S)	21 (S)	29 (S)	21 (I)	32 (S)	27 (S)	35 (S)	28 (S)	28 (S)	23 (S)	31 (S)	29 (S)

P2-76	37 (S)	30 (S)	30 (S)	19 (S)	33 (S)	22 (S)	26 (S)	18 (I)	26 (S)	20 (S)	28 (S)	22 (S)	24 (S)	26 (S)	25 (S)	31 (S)
P2-36	25 (R)	22 (S)	21 (S)	14 (I)	27 (R)	16 (S)	17 (I)	4 (R)	19 (I)	16 (S)	26 (S)	18 (S)	21 (S)	21 (S)	17 (S)	12 (R)
P2-88	42 (S)	25 (S)	26 (S)	29 (S)	38 (S)	22 (S)	32 (S)	20 (I)	30 (S)	25 (S)	33 (S)	27 (S)	30 (S)	28 (S)	26 (S)	35 (S)
P2-11	34 (S)	26 (S)	24 (S)	19 (S)	37 (S)	21 (S)	27 (S)	11 (R)	28 (S)	24 (S)	31 (S)	25 (S)	26 (S)	26 (S)	20 (S)	29 (S)
P2-154	30 (S)	22 (S)	14 (R)	13 (I)	27 (R)	14 (I)	21 (S)	7 (R)	17 (I)	15 (I)	27 (S)	16 (I)	22 (S)	13 (R)	17 (S)	22 (I)
P2-97*	36 (S)	33 (S)	31 (S)	29 (S)	35 (S)	34 (S)	32 (S)	19 (I)	35 (S)	25 (S)	32 (S)	36 (S)	31 (S)	38 (S)	23 (S)	39 (S)
P2-35	40 (S)	29 (S)	31 (S)	21 (S)	38 (S)	20 (S)	28 (S)	21 (I)	30 (S)	25 (S)	32 (S)	26 (S)	30 (S)	23 (S)	31 (S)	34 (S)
P2-34	41 (S)	28 (S)	28 (S)	22 (S)	38 (S)	26 (S)	31 (S)	17 (I)	31 (S)	26 (S)	33 (S)	28 (S)	30 (S)	30 (S)	24 (S)	33 (S)
P2-18	39 (S)	31 (S)	32 (S)	24 (S)	42 (S)	23 (S)	30 (S)	19 (I)	35 (S)	29 (S)	35 (S)	31 (S)	29 (S)	28 (S)	29 (S)	31 (S)
P2-26	35 (S)	30 (S)	29 (S)	18 (S)	41 (S)	29 (S)	30 (S)	18 (I)	32 (S)	28 (S)	34 (S)	30 (S)	27 (S)	30 (S)	25 (S)	28 (S)
P2-27	34 (S)	28 (S)	25 (S)	26 (S)	36 (S)	20 (S)	27 (S)	13 (R)	28 (S)	23 (S)	33 (S)	24 (S)	18 (I)	22 (S)	21 (S)	30 (S)
P2-173	28 (R)	23 (S)	14 (R)	10 (R)	26 (R)	15 (I)	20 (I)	6 (R)	17 (I)	15 (I)	26 (S)	15 (I)	19 (I)	19 (S)	15 (I)	12 (R)
P2-113	26 (R)	22 (S)	19 (I)	12 (R)	22 (R)	17 (S)	22 (S)	4 (R)	18 (I)	16 (S)	24 (I)	13 (R)	20 (I)	21 (S)	17 (S)	13 (R)
P2-157*	38 (S)	33 (S)	35 (S)	31 (S)	34 (S)	32 (S)	37 (S)	16 (R)	39 (S)	30 (S)	36 (S)	32 (S)	36 (S)	35 (S)	33 (S)	38 (S)
P2-38	30 (S)	22 (S)	18 (I)	15 (S)	28 (R)	13 (I)	24 (S)	8 (R)	18 (I)	10 (R)	27 (S)	19 (S)	20 (I)	16 (I)	18 (S)	20 (I)
P2-146	42 (S)	28 (S)	30 (S)	27 (S)	38 (S)	22 (S)	31 (S)	18 (I)	29 (S)	25 (S)	31 (S)	21 (S)	29 (S)	27 (S)	23 (S)	35 (S)
P2-151	38 (S)	31 (S)	27 (S)	23 (S)	41 (S)	25 (S)	30 (S)	19 (I)	33 (S)	29 (S)	33 (S)	30 (S)	28 (S)	29 (S)	22 (S)	31 (S)
P2-4	31 (S)	27 (S)	28 (S)	18 (S)	36 (S)	20 (S)	25 (S)	15 (R)	27 (S)	23 (S)	33 (S)	24 (S)	23 (S)	24 (S)	26 (S)	25 (S)
P2-168	33 (S)	25 (S)	26 (S)	20 (S)	30 (S)	18 (S)	23 (S)	12 (R)	22 (S)	17 (S)	28 (S)	20 (S)	25 (S)	26 (S)	22 (S)	27 (S)
P2-21	37 (S)	26 (S)	25 (S)	22 (S)	35 (S)	21 (S)	28 (S)	16 (R)	24 (S)	22 (S)	30 (S)	24 (S)	29 (S)	23 (S)	28 (S)	31 (S)
P2-12*	32 (S)	25 (S)	27 (S)	22 (S)	36 (S)	23 (S)	26 (S)	9 (R)	25 (S)	21 (S)	30 (S)	23 (S)	29 (S)	27 (S)	22 (S)	26 (S)
P2-68	30 (S)	23 (S)	24 (S)	14 (I)	29 (S)	15 (I)	21 (S)	10 (R)	20 (S)	18 (S)	27 (S)	19 (S)	21 (S)	14 (R)	16 (I)	22 (I)
P2-107	39 (S)	30 (S)	28 (S)	21 (S)	35 (S)	24 (S)	29 (S)	14 (R)	26 (S)	24 (S)	32 (S)	26 (S)	27 (S)	30 (S)	20 (S)	33 (S)
P2-8	41 (S)	33 (S)	31 (S)	23 (S)	39 (S)	28 (S)	32 (S)	17 (I)	30 (S)	27 (S)	34 (S)	29 (S)	30 (S)	32 (S)	28 (S)	32 (S)
P2-15	28 (R)	22 (S)	17 (I)	15 (S)	25 (R)	16 (S)	19 (I)	6 (R)	19 (I)	16 (S)	23 (I)	17 (I)	21 (S)	22 (S)	17 (S)	19 (I)
P2-103	35 (S)	28 (S)	27 (S)	26 (S)	41 (S)	30 (S)	30 (S)	17 (I)	32 (S)	29 (S)	33 (S)	30 (S)	28 (S)	31 (S)	22 (S)	30 (S)
P2-32	37 (S)	27 (S)	24 (S)	22 (S)	34 (S)	24 (S)	27 (S)	11 (R)	26 (S)	24 (S)	32 (S)	22 (S)	20 (I)	27 (S)	18 (S)	29 (S)

P2-166	30 (S)	25 (S)	26 (S)	17 (S)	33 (S)	21 (S)	23 (S)	8 (R)	24 (S)	20 (S)	30 (S)	23 (S)	19 (I)	25 (S)	29 (S)	25 (S)
P2-70*	31 (S)	33 (S)	30 (S)	24 (S)	37 (S)	26 (S)	31 (S)	18 (I)	29 (S)	25 (S)	34 (S)	30 (S)	36 (S)	33 (S)	31 (S)	34 (S)
P2-85	39 (S)	31 (S)	31 (S)	27 (S)	42 (S)	32 (S)	30 (S)	18 (I)	33 (S)	30 (S)	35 (S)	31 (S)	30 (S)	32 (S)	20 (S)	35 (S)
P2-174	32 (S)	26 (S)	29 (S)	19 (S)	36 (S)	26 (S)	25 (S)	13 (R)	30 (S)	26 (S)	34 (S)	25 (S)	24 (S)	30 (S)	21 (S)	26 (S)
P2-40	27 (R)	23 (S)	19 (I)	12 (R)	28 (R)	14 (I)	21 (S)	5 (R)	16 (R)	14 (I)	27 (S)	19 (S)	13 (R)	19 (S)	15 (I)	13 (R)
P2-117	34 (S)	26 (S)	30 (S)	23 (S)	40 (S)	28 (S)	31 (S)	17 (I)	31 (S)	26 (S)	32 (S)	30 (S)	30 (S)	32 (S)	28 (S)	30 (S)
P2-89	31 (S)	24 (S)	21 (S)	14 (I)	27 (R)	16 (S)	18 (I)	8 (R)	20 (S)	10 (R)	28 (S)	20 (S)	20 (I)	19 (S)	18 (S)	23 (S)
P2-55	29 (S)	22 (S)	22 (S)	15 (S)	25 (R)	13 (I)	21 (S)	10 (R)	19 (I)	13 (I)	26 (S)	18 (S)	19 (I)	17 (I)	17 (S)	21 (I)
P2-148*	38 (S)	30 (S)	26 (S)	31 (S)	39 (S)	28 (S)	37 (S)	21 (I)	32 (S)	28 (S)	38 (S)	30 (S)	34 (S)	29 (S)	33 (S)	38 (S)
P2-66	38 (S)	32 (S)	29 (S)	25 (S)	41 (S)	30 (S)	28 (S)	18 (I)	31 (S)	29 (S)	34 (S)	29 (S)	29 (S)	31 (S)	26 (S)	29 (S)
P2-141	36 (S)	30 (S)	28 (S)	27 (S)	40 (S)	29 (S)	30 (S)	19 (I)	32 (S)	27 (S)	33 (S)	28 (S)	28 (S)	31 (S)	29 (S)	32 (S)
P2-172	35 (S)	26 (S)	25 (S)	18 (S)	31 (S)	20 (S)	25 (S)	12 (R)	23 (S)	19 (S)	29 (S)	26 (S)	26 (S)	23 (S)	20 (S)	30 (S)
P2-135	42 (S)	31 (S)	32 (S)	22 (S)	38 (S)	27 (S)	32 (S)	21 (I)	29 (S)	26 (S)	32 (S)	31 (S)	30 (S)	30 (S)	30 (S)	32 (S)
P2-111	39 (S)	29 (S)	29 (S)	20 (S)	35 (S)	24 (S)	29 (S)	16 (R)	26 (S)	22 (S)	29 (S)	28 (S)	29 (S)	29 (S)	27 (S)	29 (S)
P2-136	34 (S)	24 (S)	24 (S)	17 (S)	33 (S)	22 (S)	25 (S)	11 (R)	25 (S)	20 (S)	31 (S)	23 (S)	26 (S)	26 (S)	25 (S)	26 (S)
P2-143	37 (S)	28 (S)	26 (S)	21 (S)	33 (S)	19 (S)	27 (S)	7 (R)	26 (S)	24 (S)	34 (S)	25 (S)	24 (S)	24 (S)	28 (S)	30 (S)
P2-137	31 (S)	27 (S)	28 (S)	29 (S)	42 (S)	26 (S)	30 (S)	18 (I)	32 (S)	30 (S)	35 (S)	20 (S)	30 (S)	31 (S)	31 (S)	23 (S)
P2-52	26 (R)	22 (S)	23 (S)	15 (S)	23 (R)	17 (S)	16 (I)	9 (R)	19 (I)	15 (I)	24 (I)	19 (S)	21 (S)	20 (S)	20 (S)	21 (I)
P2-77	28 (R)	25 (S)	24 (S)	13 (I)	33 (S)	20 (S)	19 (I)	5 (R)	18 (I)	13 (I)	27 (S)	22 (S)	20 (I)	25 (S)	17 (S)	17 (I)
P2-81	37 (S)	29 (S)	27 (S)	21 (S)	34 (S)	23 (S)	28 (S)	11 (R)	28 (S)	21 (S)	30 (S)	25 (S)	29 (S)	27 (S)	21 (S)	29 (S)
P2-120	24 (R)	23 (S)	22 (S)	16 (S)	27 (R)	15 (I)	14 (R)	6 (R)	17 (I)	9 (R)	26 (S)	16 (I)	21 (S)	14 (R)	15 (I)	13 (R)
P2-138	34 (S)	29 (S)	29 (S)	25 (S)	39 (S)	27 (S)	30 (S)	16 (R)	25 (S)	25 (S)	33 (S)	26 (S)	26 (S)	30 (S)	26 (S)	26 (S)
P2-79	37 (S)	31 (S)	32 (S)	27 (S)	40 (S)	25 (S)	28 (S)	17 (I)	32 (S)	28 (S)	35 (S)	29 (S)	28 (S)	26 (S)	22 (S)	28 (S)
P2-153	42 (S)	32 (S)	30 (S)	29 (S)	31 (S)	32 (S)	33 (S)	19 (I)	34 (S)	19 (S)	34 (S)	20 (S)	30 (S)	32 (S)	31 (S)	35 (S)
P2-134*	37 (S)	33 (S)	35 (S)	22 (S)	38 (S)	21 (S)	29 (S)	21 (I)	31 (S)	27 (S)	38 (S)	26 (S)	24 (S)	31 (S)	28 (S)	34 (S)

P2-178	37 (S)	33 (S)	31 (S)	24 (S)	42 (S)	23 (S)	31 (S)	17 (I)	31 (S)	30 (S)	35 (S)	30 (S)	27 (S)	27 (S)	30 (S)	27 (S)
P2-100	23 (R)	22 (S)	20 (I)	13 (I)	28 (R)	9 (R)	17 (I)	13 (R)	15 (R)	7 (R)	26 (S)	18 (S)	16 (I)	15 (I)	17 (S)	13 (R)
P2-39	26 (R)	24 (S)	23 (S)	11 (R)	30 (S)	13 (I)	12 (R)	8 (R)	17 (I)	10 (R)	28 (S)	20 (S)	19 (I)	17 (I)	19 (S)	15 (I)
P2-124	40 (S)	31 (S)	32 (S)	29 (S)	42 (S)	31 (S)	33 (S)	16 (R)	32 (S)	28 (S)	35 (S)	30 (S)	30 (S)	32 (S)	29 (S)	31 (S)
P2-19*	35 (S)	28 (S)	29 (S)	30 (S)	35 (S)	26 (S)	28 (S)	20 (I)	27 (S)	26 (S)	33 (S)	24 (S)	25 (S)	24 (S)	26 (S)	30 (S)
P2-133	31 (S)	24 (S)	25 (S)	17 (S)	30 (S)	18 (S)	25 (S)	12 (R)	21 (S)	17 (S)	29 (S)	18 (S)	21 (S)	16 (I)	20 (S)	23 (S)
**P2-108**	30 (S)	26 (S)	23 (S)	19 (S)	32 (S)	20 (S)	27 (S)	14 (R)	24 (S)	20 (S)	32 (S)	21 (S)	23 (S)	19 (S)	25 (S)	21 (I)
P2-123	29 (S)	22 (S)	19 (I)	15 (S)	28 (R)	10 (R)	23 (S)	9 (R)	18 (I)	18 (S)	27 (S)	19 (S)	22 (S)	14 (R)	18 (S)	22 (I)
P2-167	33 (S)	23 (S)	24 (S)	16 (S)	30 (S)	13 (I)	21 (S)	11 (R)	19 (I)	23 (S)	30 (S)	19 (S)	25 (S)	15 (I)	17 (S)	27 (S)
**P2-43**	9 (R)	6 (R)	11 (R)	12 (R)	8 (R)	13 (I)	10 (R)	0 (R)	16 (R)	11 (I)	28 (S)	5 (R)	9 (R)	14 (R)	12 (R)	13 (R)
**P2-24**	7 (R)	4 (R)	6 (R)	11 (R)	9 (R)	8 (R)	5 (R)	0 (R)	12 (R)	9 (R)	26 (S)	0 (R)	6 (R)	10 (R)	9 (R)	11 (R)
**P2-60**	12 (R)	8 (R)	9 (R)	11 (R)	15 (R)	11 (R)	13 (R)	0 (R)	15 (R)	14 (I)	26 (S)	7 (R)	8 (R)	9 (R)	10 (R)	10 (R)
**P2-181**	10 (R)	7 (R)	12 (R)	14 (I)	8 (R)	12 (R)	15 (I)	0 (R)	17 (I)	12 (I)	27 (S)	6 (R)	7 (R)	15 (I)	8 (R)	12 (R)
**P2-94**	7 (R)	9 (R)	8 (R)	12 (R)	11 (R)	15 (I)	10 (R)	0 (R)	20 (S)	10 (R)	25 (I)	5 (R)	9 (R)	14 (R)	11 (R)	9 (R)
**P2-119**	19 (R)	5 (R)	6 (R)	8 (R)	15 (R)	7 (R)	9 (R)	0 (R)	19 (I)	16 (S)	26 (S)	14 (I)	7 (R)	9 (R)	13 (I)	14 (I)
**P2-156***	10 (R)	7 (R)	5 (R)	8 (R)	4 (R)	7 (R)	11 (R)	0 (R)	14 (R)	3 (R)	19 (R)	4 (R)	10 (R)	8 (R)	6 (R)	5 (R)
P2-147	32 (S)	25 (S)	23 (S)	13 (I)	39 (S)	22 (S)	24 (S)	15 (R)	22 (S)	27 (S)	31 (S)	22 (S)	14 (I)	25 (S)	22 (S)	23 (S)
**P2-17**	11 (R)	9 (R)	10 (R)	12 (R)	14 (R)	16 (S)	14 (R)	0 (R)	17 (I)	10 (R)	25 (I)	13 (R)	12 (R)	15 (I)	12 (R)	8 (R)
**P2-92**	13 (R)	10 (R)	9 (R)	11 (R)	7 (R)	14 (I)	12 (R)	0 (R)	16 (R)	6 (R)	22 (R)	11 (R)	13 (R)	12 (R)	14 (I)	9 (R)
P2-82	37 (S)	28 (S)	26 (S)	14 (I)	34 (S)	25 (S)	22 (S)	13 (R)	25 (S)	22 (S)	33 (S)	25 (S)	16 (I)	29 (S)	21 (S)	31 (S)
P2-13	40 (S)	29 (S)	24 (S)	28 (S)	37 (S)	23 (S)	32 (S)	17 (I)	30 (S)	24 (S)	35 (S)	26 (S)	30 (S)	27 (S)	27 (S)	34 (S)
P2-45*	40 (S)	23 (S)	22 (S)	25 (S)	36 (S)	27 (S)	32 (S)	10 (R)	28 (S)	25 (S)	29 (S)	19 (S)	21 (S)	30 (S)	21 (S)	31 (S)
P2-99	30 (S)	25 (S)	24 (S)	18 (S)	33 (S)	19 (S)	23 (S)	15 (R)	23 (S)	19 (S)	29 (S)	19 (S)	25 (S)	21 (S)	21 (S)	23 (S)
P2-10	35 (S)	28 (S)	28 (S)	22 (S)	32 (S)	21 (S)	26 (S)	12 (R)	21 (S)	21 (S)	31 (S)	21 (S)	24 (S)	23 (S)	27 (S)	25 (S)
P2-23	29 (S)	23 (S)	20 (I)	16 (S)	30 (S)	18 (S)	20 (I)	5 (R)	24 (S)	17 (S)	27 (S)	20 (S)	22 (S)	20 (S)	24 (S)	27 (S)

P2-44	37 (S)	29 (S)	30 (S)	20 (S)	39 (S)	24 (S)	27 (S)	9 (R)	29 (S)	24 (S)	33 (S)	28 (S)	30 (S)	26 (S)	29 (S)	22 (I)
P2-105	31 (S)	24 (S)	24 (S)	17 (S)	33 (S)	20 (S)	24 (S)	11 (R)	26 (S)	22 (S)	30 (S)	23 (S)	25 (S)	23 (S)	25 (S)	24 (S)
P2-106	36 (S)	28 (S)	29 (S)	23 (S)	38 (S)	26 (S)	29 (S)	14 (R)	30 (S)	26 (S)	32 (S)	26 (S)	30 (S)	28 (S)	22 (S)	27 (S)
P2-72*	40 (S)	26 (S)	35 (S)	23 (S)	35 (S)	29 (S)	29 (S)	21 (I)	31 (S)	29 (S)	33 (S)	23 (S)	32 (S)	35 (S)	33 (S)	25 (S)
P2-54	40 (S)	31 (S)	30 (S)	26 (S)	39 (S)	25 (S)	31 (S)	17 (I)	33 (S)	25 (S)	31 (S)	25 (S)	30 (S)	26 (S)	28 (S)	30 (S)
P2-86	33 (S)	27 (S)	23 (S)	20 (S)	34 (S)	22 (S)	27 (S)	12 (R)	27 (S)	23 (S)	30 (S)	23 (S)	26 (S)	25 (S)	26 (S)	24 (S)
P2-22	30 (S)	25 (S)	19 (I)	15 (S)	29 (S)	17 (S)	23 (S)	10 (R)	29 (S)	17 (S)	27 (S)	18 (S)	21 (S)	21 (S)	20 (S)	21 (I)
P2-3	28 (R)	22 (S)	21 (S)	16 (S)	24 (R)	15 (I)	22 (S)	4 (R)	20 (S)	15 (I)	25 (I)	20 (S)	23 (S)	17 (I)	18 (S)	13 (R)
P2-61*	38 (S)	30 (S)	25 (S)	18 (S)	35 (S)	21 (S)	28 (S)	5 (R)	30 (S)	26 (S)	32 (S)	24 (S)	27 (S)	28 (S)	29 (S)	27 (S)
P2-175	39(S)	27 (S)	27 (S)	21 (S)	37 (S)	21 (S)	30 (S)	16 (R)	28 (S)	22 (S)	33 (S)	27 (S)	29 (S)	23 (S)	27 (S)	29 (S)
P2-59	29 (S)	26 (S)	25 (S)	17 (S)	31 (S)	20 (S)	22 (S)	11 (R)	22 (S)	18 (S)	26 (S)	20 (S)	23 (S)	24 (S)	26 (S)	28 (S)
P2-65	27 (R)	23 (S)	20 (I)	14 (I)	28 (R)	13 (I)	21 (S)	6 (R)	21 (S)	16 (S)	27 (S)	14 (I)	20 (I)	19 (S)	17 (S)	18 (I)
P2-95	24 (R)	22 (S)	21 (S)	15 (S)	26 (R)	18 (S)	19 (I)	8 (R)	20 (S)	15 (I)	22 (R)	12 (R)	18 (I)	22 (S)	15 (I)	13 (R)
P2-139	37 (S)	29 (S)	28 (S)	21 (S)	34 (S)	22 (S)	28 (S)	13 (R)	25 (S)	23 (S)	31 (S)	23 (S)	26 (S)	26 (S)	24 (S)	31 (S)
P2-101	41 (S)	30 (S)	32 (S)	27 (S)	39 (S)	27 (S)	32 (S)	19 (I)	30 (S)	25 (S)	32 (S)	28 (S)	30 (S)	29 (S)	31 (S)	35 (S)
P2-163	34 (S)	25 (S)	28 (S)	19 (S)	37 (S)	23 (S)	24 (S)	12 (R)	28 (S)	22 (S)	30 (S)	25 (S)	29 (S)	25 (S)	29 (S)	26 (S)
P2-165	30 (S)	27 (S)	23 (S)	17 (S)	32 (S)	20 (S)	22 (S)	10 (R)	26 (S)	18 (S)	28 (S)	21 (S)	24 (S)	23 (S)	21 (S)	24 (S)
P2-115*	38 (S)	32 (S)	30 (S)	28 (S)	33 (S)	35 (S)	38 (S)	21 (I)	36 (S)	28 (S)	40 (S)	34 (S)	31 (S)	37 (S)	28 (S)	39 (S)
P2-6	36 (S)	32 (S)	30 (S)	21 (S)	39 (S)	21 (S)	26 (S)	14 (R)	31 (S)	27 (S)	31 (S)	29 (S)	29 (S)	22 (S)	30 (S)	29 (S)
P2-126	29 (S)	24 (S)	23 (S)	13 (I)	31 (S)	16 (S)	21 (S)	8 (R)	25 (S)	19 (S)	28 (S)	20 (S)	23 (S)	20 (S)	23 (S)	22 (I)
P2-177	32 (S)	28 (S)	25 (S)	19 (S)	35 (S)	24 (S)	25 (S)	11 (R)	28 (S)	24 (S)	34 (S)	24 (S)	27 (S)	25 (S)	27 (S)	27 (S)
P2-176	26 (R)	22 (S)	20 (I)	11 (R)	28 (R)	14 (I)	22 (S)	5 (R)	18 (I)	14 (I)	26 (S)	16 (I)	21 (S)	16 (I)	17 (S)	12 (R)
P2-69	40 (S)	32 (S)	30 (S)	25 (S)	37 (S)	22 (S)	30 (S)	20 (I)	27 (S)	25 (S)	30 (S)	26 (S)	29 (S)	24 (S)	28 (S)	32 (S)
P2-114	33 (S)	27 (S)	26 (S)	18 (S)	39 (S)	25 (S)	27 (S)	13 (R)	29 (S)	27 (S)	32 (S)	24 (S)	30 (S)	27 (S)	31 (S)	27 (S)

P2-158*	40 (S)	39 (S)	28 (S)	26 (S)	37 (S)	25 (S)	40 (S)	21 (I)	28 (S)	20 (S)	42 (S)	38 (S)	30 (S)	32 (S)	39 (S)	40 (S)
P2-25	42 (S)	29 (S)	31 (S)	27 (S)	40 (S)	28 (S)	33 (S)	17 (I)	32 (S)	24 (S)	35 (S)	29 (S)	30 (S)	30 (S)	24 (S)	34 (S)
P2-28	31 (S)	24 (S)	22 (S)	16 (S)	29 (S)	17 (S)	21 (S)	14 (R)	22 (S)	17 (S)	27 (S)	18 (S)	22 (S)	20 (S)	30 (S)	25 (S)
P2-155	37 (S)	30 (S)	28 (S)	22 (S)	35 (S)	20 (S)	28 (S)	12 (R)	26 (S)	20 (S)	29 (S)	23 (S)	27 (S)	22 (S)	22 (S)	31 (S)
P2-162	35 (S)	29 (S)	25 (S)	21 (S)	34 (S)	19 (S)	24 (S)	9 (R)	25 (S)	21 (S)	31 (S)	22 (S)	25 (S)	23 (S)	27 (S)	29 (S)

Note: AMP, ampicillin; FOX, cefoxitin; CLI, clindamycin; GEN, gentamicin; AMX, amoxicillin; DOX, doxycycline; LCM, lincomycin; CAZ, ceftazidime; RIF, rifampin; SXT, Sulfmethoxazole-Trimethoprim; LZD, linezolid; AZM, azithromycin; CFO, ceftriaxone; TET; tetracycline; NOR, norfloxacin; ERY, erythromycin; mm, millimeter; S, sensitive; I, intermediate; R, resistant; * human samples; MRSA in bold font.
