# Supplementary figures and images for: Comprehensive Molecular and Epidemiological Characterization of Staphylococcus aureus Isolated from Bovine Mastitis in Water Buffalo of the Peshawar Division, Khyber Pakhtunkhwa, Pakistan
Source: Pathogens. 2025 Jul 25;14(8):735. doi: 10.3390/pathogens14080735 (PMC12388927; doi:10.3390/pathogens14080735)

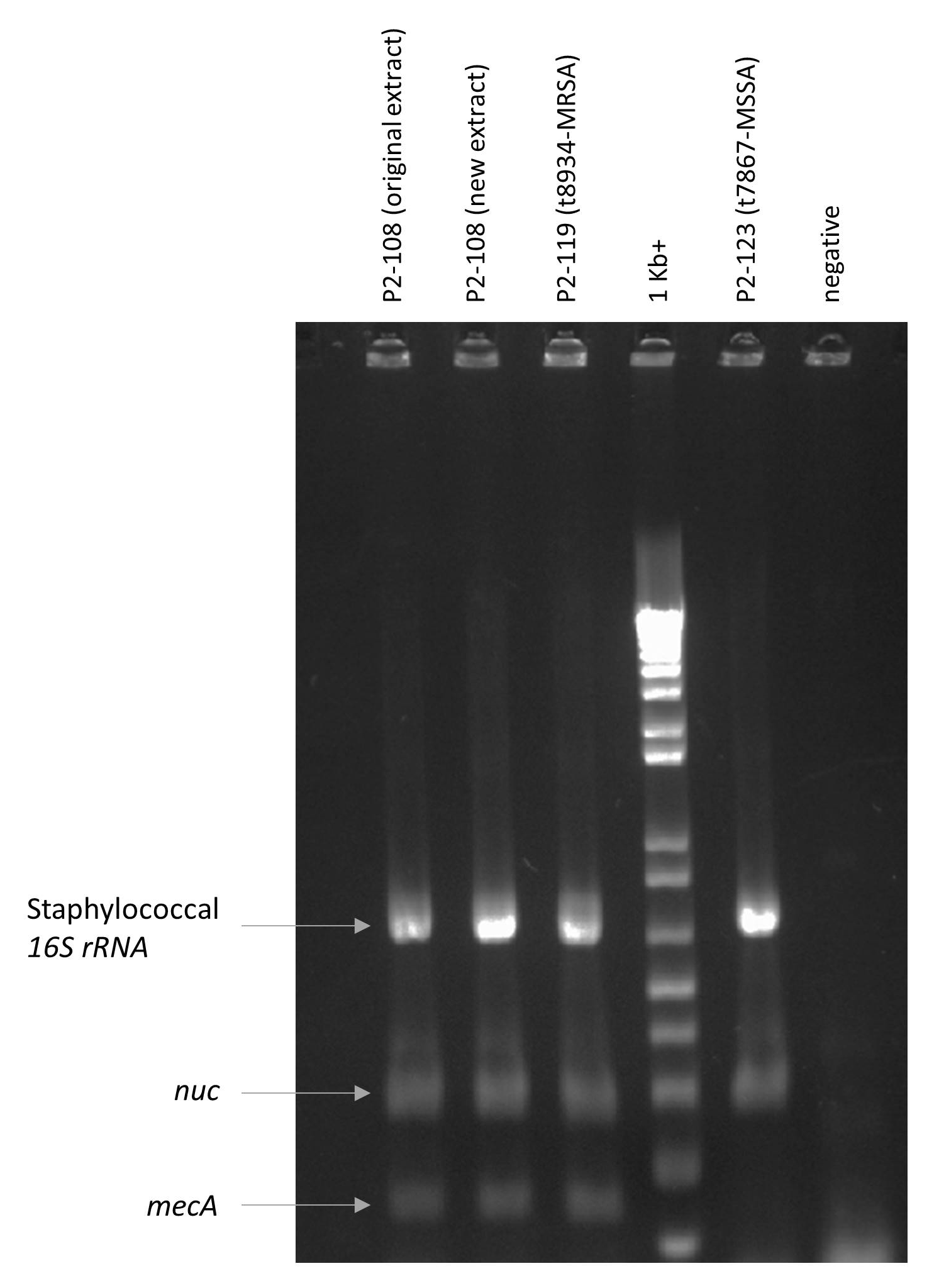

Supplement: Supplementary file 1 [file pathogens-14-00735-s001.zip › Figure S1.tiff]

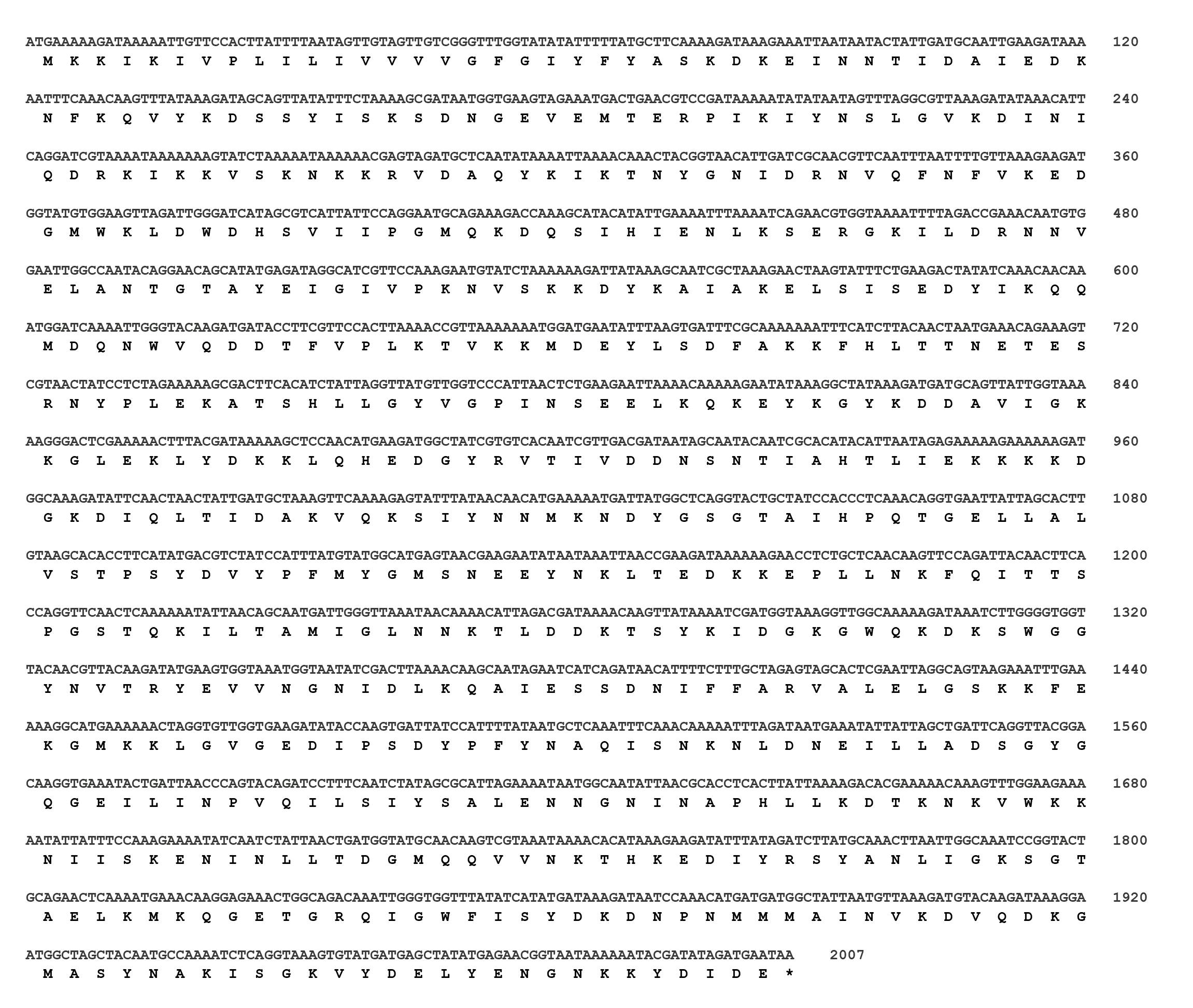

Supplement: Supplementary file 1 [file pathogens-14-00735-s001.zip › Figure S2.tiff]
